# Extrapulmonary Neuroendocrine Carcinomas: Current Management and Future Perspectives

**DOI:** 10.3390/jcm12247715

**Published:** 2023-12-15

**Authors:** Sara Stumpo, Maria Giovanna Formelli, Irene Persano, Elena Parlagreco, Eleonora Lauricella, Maria Grazia Rodriquenz, Luigi Pio Guerrera, Ina Valeria Zurlo, Davide Campana, Maria Pia Brizzi, Mauro Cives, Anna La Salvia, Giuseppe Lamberti

**Affiliations:** 1 Department of Medical and Surgical Sciences (DIMEC), Alma Mater Studiorum–University of Bologna, Via Zamboni 33, 40126 Bologna, Italy; sara.stumpo@studio.unibo.it (S.S.); maria.formelli@studio.unibo.it (M.G.F.); davide.campana@unibo.it (D.C.); giuseppe.lamberti8@unibo.it (G.L.); 2 Medical Oncology, AO S. Croce e Carle, 12100 Cuneo, Italy; irene.persano@gmail.com (I.P.); elena.parlagreco@edu.unito.it (E.P.); 3 Medical Oncology Unit, Azienda Ospedaliero-Universitaria Consorziale Policlinico di Bari, 70124 Bari, Italy; ele.lauricella@gmail.com (E.L.); mauro.cives@uniba.it (M.C.); 4 Oncology Unit, Ospedale IRCCS Casa Sollievo della Sofferenza, 71013 San Giovanni Rotondo, Italy; grazia.rodriquenz@gmail.com; 5 Division of Medical Oncology, Department of Precision Medicine, University of Campania “Luigi Vanvitelli”, 80131 Naples, Italy; luigipio.guerrera@unicampania.it; 6 Sarcomas and Rare Tumors Unit, Istituto Nazionale Tumori, IRCCS-Fondazione “G. Pascale”, 80131 Naples, Italy; 7 Medical Oncology Unit, Vito Fazzi Hospital, 73100 Lecce, Italy; valeriazurlo26@gmail.com; 8 Medical Oncology Unit, IRCCS Azienda Ospedaliero-Universitaria di Bologna, Via P. Albertoni 15, 40138 Bologna, Italy; 9 Department of Oncology, A.O.U. San Luigi Gonzaga Hospital, 10043 Orbassano, Italy; brizzimariapia@gmail.com; 10 Department of Interdisciplinary Medicine, University of Bari “Aldo Moro”, 70121 Bari, Italy; 11 National Center for Drug Research and Evaluation, National Institute of Health (ISS), 00161 Rome, Italy

**Keywords:** extrapulmonary neuroendocrine carcinomas, chemotherapy, gastroenteropancreatic tract, gynecologic tract, genitourinary tract

## Abstract

Neuroendocrine carcinomas (NECs) are poorly differentiated and highly aggressive epithelial neuroendocrine neoplasms. The most common primary site is the lung, but they may arise in every organ. Approximately 37% of extrapulmonary NECs (EP-NECs) occur in the gastroenteropancreatic (GEP) tract, followed by the genitourinary (GU) system and gynecological tract. As a result of their rarity, there is scant evidence to guide treatment recommendations, and a multidisciplinary approach is essential for the management of such patients. Platinum-based chemotherapy currently represents the standard of care for EP-NECs of any site, mirroring the management of small-cell lung cancer (SCLC), but further approaches are still under investigation. Indeed, ongoing trials evaluating targeted therapies, immune checkpoint inhibitors (ICIs), and radionuclide therapy could provide potentially breakthrough therapeutic options. Given the relative dearth of evidence-based literature on these orphan diseases, the aim of this review is to provide an overview of the pathology and current treatment options, as well as to shed light on the most pressing unmet needs in the field.

## 1. Introduction

Neuroendocrine carcinomas (NECs) are a rare and heterogeneous entity that belongs to the family of neuroendocrine neoplasms (NENs) [1,2]. According to the 2022 World Health Organization (WHO) classification of endocrine and neuroendocrine tumors [3,4], NECs are defined as poorly differentiated, highly aggressive (G3) epithelial NENs, composed of cells characterized by marked nuclear/cellular atypia and severe nuclear molding, but broadly retaining neuroendocrine markers (of note, the pan-cytokeratin stain should be positive, even focally, as their origin is epithelial) [5]. By definition, the Ki-67 proliferation index is ≥20%, usually being above 50%.

Irrespective of their primary site of origin, NECs are commonly subclassified as small or large cell types (SCNEC and LCNEC, respectively), thus suggesting the possible presence of pathogenetic and clinical differences between the two entities, as already demonstrated for lung primaries [3,4].

NECs arise from neuroendocrine cells, which can be arranged in glands (e.g., pituitary and parathyroid), endocrine islets embedded within glandular tissue (e.g., thyroid and pancreas) or scattered/interspersed among other epithelial cells (e.g., respiratory, digestive, genitourinary tracts, thymus, and skin) [6,7]. Derivation from stem cells as well as from trans-differentiated epithelial cells has been also suggested.

This may explain why NECs may develop in any organ, with common histology features (small-cell or large-cell morphology, poor differentiation, marked atypia, abundant necrosis, and frequent, often atypical mitoses) and a similar clinical behavior (high propensity for distant metastases and poor prognosis) [3].

Besides the lung, which is the most common primary site, NECs that arise outside of this location are referred to as extrapulmonary NECs (EP-NECs), and these are most commonly found in the gastroenteropancreatic (GEP) tract (about 37% of cases), followed by the genitourinary (GU) tract (about 17%) and gynecological (GY) tract (about 10%) [8,9]. EP-NECs are generally non-functioning: their clinical presentation is often the result of a combination of site-specific symptoms and the typical symptoms of advanced/metastatic cancers (weight loss, weakness, etc.) [1].

Opposite of well-differentiated neuroendocrine tumors (NETs), NECs are characterized by the frequent presence of inactivating *TP53* or *RB1* gene alterations, regardless of the origin. Indeed, inactivating *RB1* gene mutations or the loss of Rb protein expression have been reported in >90% of SCNECs and in 50 to 60% of LCNECs. Similarly, *TP53* gene mutations or abnormal protein expression have been reported in up to 95% of NECs [3,10]. Additional site-specific genetic alterations have also been described and may be helpful to detect the primary site of origin [11]. Despite *TP53* and *RB1* alterations being non-targetable, targetable driver alterations such as *NTRK*, *RET*, and *BRAF* [12,13,14,15] can be found in EP-NECs and should be sought after.

For all the above reasons, the treatment of EP-NECs is particularly challenging, and the little available evidence comes from weak sources, mainly limited by retrospective and small-sample-size studies. Additionally, EP-NECs are seldom included in NEN clinical trials given their rarity, further limiting the production of solid prospective evidence in these patients. EP-NEC treatment recommendations are usually extrapolated and adapted from pulmonary high-grade NECs, especially small-cell lung cancer (SCLC), since this is the most common type of NEC [16].

Given the relative dearth of evidenced-based literature on these orphan diseases, this review aims to provide an overview of the pathology and current treatment options, as well to shed light on the most pressing unmet needs in EP-NECs.

## 2. Materials and Methods

We reviewed all prospective and retrospective studies, case reports, and review articles published up to June 2023 in PubMed. Data were extracted from the text and from the tables of the manuscripts. The keyword search used included “neuroendocrine carcinomas”, “gastroenteropancreatic”, “colorectal”, “esophageal”, “stomach”, “gynecologic”, “cervical”, “endometrial”, “ovarian”, ”vagina”, “vulva”, “genito-urinary”, “renal”, “prostatic”, “bladder”, “small cell”, “large cell”, and “extrapulmonary”.

## 3. GEP-NECs

### 3.1. Epidemiology and Staging

GEP-NECs account for about one third of all NEC diagnoses and are often managed similarly to pulmonary NECs [9]. While smoking is the key risk factor for pulmonary NECs, most patients with GEP-NECs do not have any history of smoking [17]. Along the same line, while brain metastases are reported in about 20% of pulmonary NECs, they are very rare in GEP-NECs, occurring in less than 2% of cases [18].

Within the GEP tract, the most common sites of primary GEP-NEC are the colon (approximately 25%), pancreas (20%), stomach and esophagus (more than 20% together), and rectum (just above 10%) [19]. Up to 85% of patients have a stage IV disease at diagnosis, with a median overall survival (OS) of about 12 months [20]. In fact, prognosis is slightly better for GEP than pulmonary NECs and differs by primary tumor location (the OS being longer in small-bowel primary tumors), but it remains poor overall [21]. As for other EP-NECs, a CT scan of the chest and abdomen is required, while brain imaging is not routinely indicated in asymptomatic patients. Functional imaging with 18F-FDG-PET might be useful to complete staging, while 68Ga-DOTA-peptide PET imaging is not recommended outside of clinical trials.

### 3.2. Management of GEP-NECs

**Figure 1:**
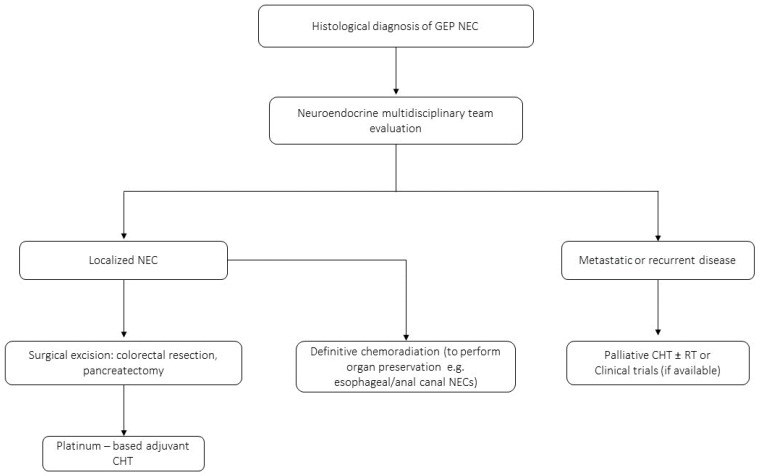
Proposed clinical management algorithm for GEP-NEC. CHT: chemotherapy. RT: radiotherapy.

Although the role of surgery remains controversial for NECs (especially because preoperative diagnosis of a localized NEC is uncommon), international guidelines suggest surgery for patients with a localized NEC, strongly recommending a careful multidisciplinary assessment of individual cases (Figure 1) [1,22]. In fact, in some patients, perioperative treatment with chemotherapy (eventually associated with radiotherapy) may be considered, but every decision should be individualized [19]. Definitive chemoradiation is usually the preferred approach for anatomical sites where surgical resection has a high risk of morbidity, such as esophageal or anal canal NECs [23,24]. Large retrospective series suggested that surgery in carefully selected cases significantly improves survival regardless of the primary tumor site and other perioperative therapies [19,25]. A recent retrospective study investigated the effect of surgical resection in one of the most common sites of origin (the colorectal tract), including 1208 NEC patients from the National Cancer Database. The analysis showed a beneficial effect of surgery with a better median OS in patients undergoing surgery compared to those managed without resection [26]. However, an earlier retrospective study showed opposite results highlighting that the resection of the primary tumor was not associated with a survival improvement in localized colorectal NECs [27]. Overall, the evidence is scarce mainly due to the retrospective nature of these studies as well as their limited sample size [28].

As previously mentioned, conservative management with chemoradiotherapy may be particularly important in some special anatomical sites where the diagnosis of NEC still is a major challenge in terms of disease control and organ preservation. Chemo-radiation is the preferred treatment to obtain organ preservation in anorectal NECs and is usually associated with manageable side effects (the most common being proctitis) [29]. In addition, few studies suggest definitive chemoradiation may also be useful for esophageal primaries. Since the most common reason of radiotherapy failure in the treatment of NECs is distant progression, the combination of local treatments with systemic therapy is of paramount importance. Cisplatin or carboplatin + etoposide are often used in combination with radiotherapy [30]. A retrospective analysis from the SEER (Surveillance Epidemiology and End Results) database showed a significant survival advantage with the use of radiation therapy in the setting of rectal SCNECs [31], in contrast to other studies demonstrating that chemotherapy with or without radiation obtained a similar outcome to surgery in patients with localized anorectal NECs [32]. This difference can possibly be explained by the limited number of cases undergoing exclusive chemotherapy, hindering any definitive conclusions on the potential benefit of radiotherapy in this setting.

Peptide receptor radionuclide therapy (PRRT) with 177Lutetium-DOTATATE is currently approved in the treatment of well-differentiated metastatic G1-2 GEP-NETs [33]. In contrast, PRRT is generally not recommended for GEP-NECs because of the low-to-absent expression of somatostatin receptors [34,35]. An Italian group investigated the outcomes of PRRT in 33 patients with advanced GEP-NENs, positive somatostatin receptor imaging, and a Ki-67 proliferation index between 15% and 70%, and found that patients with a Ki-67 index of ≥35%, achieved worse response rates and a shorter progression-free survival (PFS) as compared to patients with tumors with a lower proliferation index [36]. Similarly, one large retrospective multicenter study demonstrated disappointing survival outcomes of PRRT in patients with GEP-NECs as compared to those with well-differentiated G3 NENs [37].

As the role of radical surgery in GEP-NECs is still being defined, the role of adjuvant chemotherapy also remains controversial, and decisions need to be individualized based on the tumor site and histological features and after multidisciplinary team assessment [1]. Similar to pulmonary LCNECs where weak evidence of a higher survival in patients receiving adjuvant therapy after radical surgery has been reported [38], the Society of Surgical Oncology reported a non-statistically significant trend towards better survival in a retrospective series of 18 patients with GEP-NECs who received adjuvant therapy after surgical resection [39]. The small sample size and retrospective design of the study hinder reliable conclusions. The NEONEC trial is currently investigating the activity of adjuvant platinum-based chemotherapy in patients who underwent surgery for digestive NECs, and the results are eagerly awaited (NCT04268121) [8].

When metastatic, NECs tend to initially respond to chemotherapy, with reported objective response rates (ORRs) of up to 70% [16,40]. Since the early 1990s, cisplatin + etoposide has been considered the reference treatment for NECs, including GEP-NECs, based on its activity in SCLC [16]. In the last decade, the largest reported cohort of advanced patients with EP-NECs, defined as NENs with a Ki-67 ≥ 20%, is from the Nordic group [35]. They retrospectively analyzed data from 205 patients with a GI NEC, confirming the effectiveness of the platinum-based regimen, but at the same time suggesting a lower response rate compared to what was previously reported. In addition, their results indicated that in GI NECs, cisplatin could probably be replaced by the less toxic carboplatin, as the efficacy of the two compounds was comparable. Notably, GEP-NECs with a Ki-67 > 55% had a better response to platinum-based chemotherapy and shorter median survival than NECs characterized by lower proliferative activity (20–55%). More recently, a Chinese phase II study compared the activity of the standard regimen (cisplatin + etoposide) to the combination of cisplatin + irinotecan as first-line treatment in 66 patients with advanced GEP-NECs [41]. They also evaluated the different toxicity profiles of the two associations. The irinotecan arm was not inferior to the etoposide arm in terms of ORR, but different safety profiles emerged. The incidence of grade 3 and 4 neutropenia was significantly higher in the etoposide arm while non-hematological toxicities, in particular GI toxicities, were relatively more frequent in the irinotecan arm. Despite the good ORR observed, the responses were commonly short in duration, lasting on average only 9 months.

At progression, one option is represented by rechallenging with platinum + etoposide. This combination is usually proposed when the relapse occurs at least 60–90 days after the end of the previous therapy (treatment-free interval, TFI), mirroring SCLC practice [42]. Otherwise, different regimens including FOLFIRI [43], FOLFOX [44] or temozolomide-based chemotherapies have been evaluated [45]. The relative studies share a retrospective design and a small sample size (ranging from 20 to 30 patients), and an ORR between 29% and 33% and median PFS of 4–6 months have been reported.

Several drugs and regimens, such as dacarbazine, topotecan, liposomial irinotecan + 5-fluorouracil, docetaxel, and the addition of bevacizumab, a humanized monoclonal antibody against the vascular endothelial growth factor (VEGF), to FOLFIRI, failed to prove beneficial in GEP-NEC patients [46,47,48,49]. Nevertheless, the SENECA study, a randomized phase II trial of CAPTEM vs. FOLFIRI in lung and GEP-NECs, completed enrollment and the results are awaited soon (NCT03387592) [50].

The interest around immunotherapy with immune checkpoint inhibitors (ICIs) and targeted therapies in GEP-NECs is growing given the potential for better-tolerated treatment alternatives and/or long-lasting responses. Single-agent ICIs have not been proven effective in NECs, especially in unselected populations. However, currently there is no biomarker to predict the response to ICIs in patients with GEP-NECs. Among the known predictive factors of the response to ICIs, PD-L1 expression is commonly weak and restricted to tumor-infiltrating lymphocytes (TILs) rather than tumor cells in GEP-NECs [51], while a high microsatellite instability status and a high tumor mutational burden have been observed in less than 10% of GEP-NECs [51,52].

To overcome the primary resistance to single-agent ICIs, various combination strategies have been investigated. The combination of cytotoxic T-lymphocyte antigen 4 (CTLA-4) and programmed death 1 (PD-1) blockers was hypothesized to have synergistic effects on antitumor immune response and to increase the response rates in patients. In the DUNE study, the combination of durvalumab (PD-L1 inhibitor) + tremelimumab (CTLA-4 inhibitor) was tested in four NEN cohorts including some cases of GEP-NECs, demonstrating a modest response rate. Among the ten patients who survived for more than a year, seven had poorly differentiated NECs but no significant correlation was found between baseline molecular or clinical biomarkers and the efficacy in this subgroup [53]. Likewise, the NIPINEC study investigated the role of the dual ICI PD-1/CTLA-4 (nivolumab + ipilimumab) in GEP-NECs [54]. This was a randomized noncomparative trial that included 92 patients with GEP-NECs progressing to one or two previous systemic treatment lines including platinum-based chemotherapy. The results showed a modest activity of the regimen in aggressive and heavily pretreated NECs. Another possibility to implement the effect of the ICIs is obviously the combination with chemotherapy, as already demonstrated in SCLC. In clinical practice, ICIs combined with chemotherapy already constitute a standard treatment modality for patients with SCLC [55]. Similarly, in the phase II NICE-NEC trial, 26 patients with GEP-NECs (or unknown origin)underwent treatment with a combination of nivolumab + carboplatin + etoposide followed by a maintenance therapy with nivolumab. The primary endpoint was a 12-month OS rate and the results were quite encouraging [56]. Specifically, the 12-month OS rate was 46.7% vs. 59.3% for Ki-67 ≤ 55 and >55, respectively, and 58.3% vs. 54.7% for well- and poorly differentiated, respectively.

Data supporting the use of targeted therapies in GEP-NECs are still missing, as targetable driving genomic alterations are uncommonly seen. One of the most common molecular alterations, especially in colorectal NECs, is KRAS mutation. Recently, small molecules that specifically inhibit KRAS^G12C^ demonstrated activity in patients with advanced solid tumors harboring this mutation [57,58], but this has not been tested in patients with KRAS^G12C^-mutated GEP-NECs. In the mitogen-activated kinase pathway (MAPK), BRAF^V600E^ mutation can be observed in colorectal and pancreatic NECs. This alteration can be targeted by BRAF inhibitors with or without MEK inhibitors. The combination of dabrafenib and trametinib, a BRAF and an MEK inhibitor, respectively, has been evaluated in an open-label basket trial, showing a promising overall response rate in 29 patients including two cases with mixed ductal/adeno-neuroendocrine carcinomas and two with colorectal NECs [15]. In addition, a phase II basket trial evaluating the role of the BRAF inhibitor vemurafenib in non-melanoma BRAF^V600E^ mutant solid tumors, showed an encouraging PFS for two patients with NECs [59]. These results demonstrate a potential benefit of BRAF/MEK inhibition in patients with GEP-NECs with BRAF^V600E^ mutation.

NENs, and especially pancreatic NENs are highly vascularized tumors as a result of the overexpression of pro-angiogenic factors, including VEGF receptor and platelet-derived growth factor receptor (PDGFR) [60]. A phase II trial evaluated the clinical activity of sunitinib, a multi-targeted tyrosine kinase inhibitor (TKI) with anti-angiogenic activity, in 26 patients with GEP-NENs (20 GEP-NECs and 6 G3 NETs), mostly of pancreatic origin, and reported an ORR of 58% [61]. Of the 20 poorly differentiated GEP-NECs, 10 patients presented a radiologic response by RECIST 1.1 criteria.

In another phase II trial, 22 treatment-naïve patients received a combination of capecitabine + oxaliplatin + CPT-11 (CAPOXIRI) + bevacizumab, followed by maintenance treatment with pazopanib (anti-angiogenic TKI) + capecitabine. The combination demonstrated promising efficacy with a median PFS of 13 months, median OS of 29 months and ORR of 47.4% for large- and small-bowel neuroendocrine carcinomas. Furthermore, predictable toxicities were observed with this regimen [62].

## 4. GY-NECs

### 4.1. Epidemiology and Staging

High-grade neuroendocrine carcinomas of the gynecological tract (GY-NECs) are rare and highly aggressive tumors, which account for about 2% of gynecologic malignancies and less than 1% of all NECs [9,63]. Overall, GY-NECs show aggressive behavior with a high recurrence rate in localized stages or the early development of metastases and, therefore, poor prognosis.

According to the updated fifth edition of the WHO classification of gynecologic NENs, grade 3 tumors are poorly differentiated high-grade NECs that are further classified into SCNECs and LCNECs [64].

At pathology, SCNECs and LCNECs show the same histological features as their counterparts in other sites. SCNECs are usually hypercellular, characterized by monotonous diffuse pattern of growth, high mitotic activity (>10 mitoses/10 HPF), apoptotic bodies, and widespread areas of necrosis, with early lymph–vascular invasion. Instead, LCNECs mostly show a solid pattern of growth, including insular, trabecular, and glandular patterns, with high mitotic activity, and frequent vascular involvement. In addition, up to one third of GY-NECs are associated with an intraepithelial or invasive non-neuroendocrine component, often a squamous neoplasia [64,65]. Despite the typical morphology, in some cases diagnosis can be challenging, especially for LCNECs; therefore, immunohistochemistry (IHC) for neuroendocrine markers including chromogranin A (CgA), CD56, synaptophysin, and protein gene product 9.5 (PGP9.5) may be required for diagnosis. Synaptophysin and CD56 are the most sensitive markers, although CD56 is non-specific. Furthermore, INSM1 (insulinoma-associated protein 1) expression is more specific than chromogranin A, CD56, and synaptophysin in GY-NECs, but less sensitive than synaptophysin, and not significantly different among SCNECs, LCNECs, and mixed SCNECs–LCNECs [66].

While the ovary is the most common site of origin for NETs of the gynecological tract [11], GY-NECs most frequently arise from the cervix (54%), most commonly as SCNECs, followed by the endometrium (24%), the ovary (16%), the vagina (5%), and the vulva (1%) [67].

Clinical presentation of GY-NECs usually include weight loss, localized pain and pressure, vaginal bleeding and/or discharge, ascites, and symptoms depending of the sites of the metastases [68]. GY-NECs show a higher frequency of lymph–vascular invasion and early distant metastasis and recurrence than non-neuroendocrine gynecologic cancers. Therefore, the Society of Gynecologic Oncology (SGO) and NCCN guidelines recommend the first evaluation with chest, abdomen, and pelvis TC and/or 18F-FDG-PET/TC, with additional brain imaging if neurological symptoms are present or metastases are suspected.

### 4.2. NECs of the Cervix

NENs of the cervix account for about 0.9% to 1.5% of all cervical tumors, with SCNECs being the most common subtype [69].

Similar to non-neuroendocrine cervical cancer, most SCNECs and LCNECs of the cervix are caused by human papillomavirus (HPV) infection, mainly types 16 and 18, with a predominance of HPV18 over HPV16 among SCNECs [70]. The causative role of HPV infection in NECs of the cervix is reflected by the high p16 IHC positivity rate reported in these tumors [64], as opposed to what is observed in cervical NETs [69], and suggests that HPV vaccination could prevent up to 95% of cervical NECs [71].

The top mutated genes in GY-NECs of the cervix are PIK3CA, KRAS, and TP53 in 18%, 14%, and 11% of cases, respectively [72]. Moreover, the presence of genetic alterations in the MAPK, p53/BRCA, and PI3K/AKT/mTOR pathways suggests that targeted therapies might be potentially useful in a proportion of cases [73].

Cervical NENs are staged using the same 2018 FIGO system as for non-neuroendocrine cervical cancers [74,75]. NECs are more likely to present at later FIGO stages, and to have lymph node involvement at diagnosis, as compared to non-neuroendocrine carcinomas [76]. The median OS for patients with SCNECs of the cervix is less than 2 years from diagnosis [76], with a five-year OS rate of 20–50% in stage I–II tumors, which falls to 2–15% for later-stage tumors [74].

**Figure 2:**
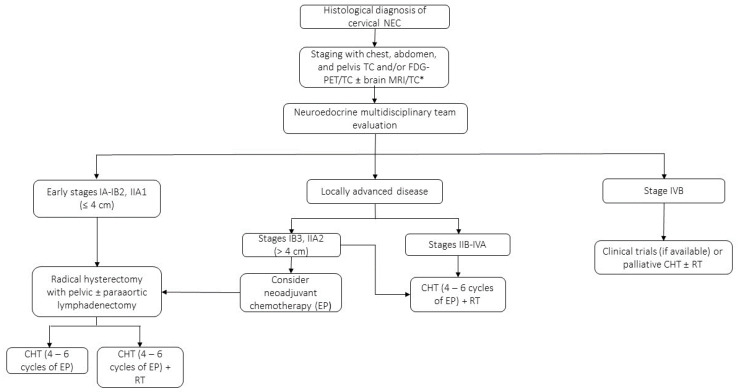
Proposed clinical management algorithm for cervical NECs based on FIGO staging system (2018). EP: etoposide + cisplatin or carboplatin. Cisplatin (60–80 mg/m^2^ on day 1 every 3 weeks) or carboplatin (area under the curve of 5 mg/mL/min on day 1 every 3 weeks) + etoposide (100–120 mg/m^2^ on day 1–3 every 3 weeks). RT: 40–45 Gy external beam radiation ± 40–45 Gy vaginal brachytherapy. * Brain evaluation in cases of symptoms or suspected metastases.

Because of the lacking prospective data, recommendations for the treatment of patients with GY-NECs are extrapolated from SCLC treatment. As such, the standard treatment algorithm for cervical NECs is based on a combination of surgery, platinum-based chemotherapy, and radiotherapy (Figure 2) [77]. Both the SGO and the Gynecologic Cancer Intergroup (GCIG) recommend radical hysterectomy and pelvic lymphadenectomy followed by adjuvant chemotherapy with platinum + etoposide for early-stage disease (tumors ≤4 cm) and negative nodes on imaging (up to FIGO stage IIA1, approximately 50% of cases [78]), with consideration for additional radiotherapy, as women who received multimodality treatment can achieve long-term survival [79]. The role of surgery in early-stage NECs of the cervix has been strengthened by the results of a recent study, which included data of 1288 patients collected from the SEER database and from a Chinese multi-institutional registry [80]. Taking into consideration the limitations and biases of surgical studies conducted on retrospective series, surgery might improve the outcomes of patients with locally advanced or stage IB3-IIA2 NECs of the cervix, although in these cases surgery is not currently routinely recommended by guidelines. On the other hand, post-operative radiotherapy after radical hysterectomy in women with early-stage NECs of the cervix may reduce pelvic recurrences but has not been proven to improve the overall recurrence or death rates, mainly due to the high rate of distant extra-pelvic recurrences [81]. Both adjuvant cisplatin + etoposide and vincristine + cyclophosphamide + doxorubicin (CAV regimen) were associated with improved survival compared to other schedules, but considering the toxicity profile of the two regimens, platinum + etoposide is preferred over CAV in the adjuvant setting [82].

When it comes to locally advanced disease (FIGO stage IIA2-IV, approximately 49% of cases) [78] or non-surgical candidates, SGO guidelines recommend concomitant platinum + etoposide chemotherapy and radiation (to pelvis or up to para-aortic lymph nodes) [79], which yield a 3-year OS rate of 60%, and a potential cure rate of 45%. The most relevant prognostic factor in this setting is the stage at diagnosis [83].

Approximately 23% of NECs of the cervix are diagnosed at stage IV (compared with 8% of squamous-cell carcinomas) [76]. In addition, even after multimodality treatment, NECs have a high rate of recurrence, with the most common sites for first recurrence being the lungs (38%), liver (34%), and peritoneal carcinomatosis (25%). Brain recurrence is only observed in patients with concurrent lung or liver metastases [84]. The standard first-line treatment for advanced treatment-naïve NECs of the cervix is chemotherapy with platinum + etoposide for 4–6 cycles [79]. At disease progression, a rechallenge with the same chemotherapy scheme can be considered in patients with a TFI of at least 60–90 days, mirroring the management of SCLC [42]. In patients who have already received a platinum-containing chemotherapy regimen or who have relapsed with a TFI of 60–90 days, single-agent chemotherapy with topotecan, irinotecan, paclitaxel, or docetaxel is recommended. A single-institution experience also reports an improvement in PFS, but not OS, using a combination of topotecan + paclitaxel with bevacizumab [85]. This schedule has been investigated in non-neuroendocrine cervical cancers in the Gynecology Oncology Group 240 phase III study [86] and is based on the rationale that over 95% of cervix SCNECs overexpress VEGF [84,87]. Nevertheless, the available evidence is not adequate to recommend this regimen, which might be considered in select cases.

Despite the aggressive treatment strategies involving radical surgery, chemotherapy, and radiotherapy, women with SCNECs of the cervix have a dismal prognosis with a median OS of less than 2 years from diagnosis [76], especially at recurrence [69]. Innovative therapeutic approaches, such as ICIs or targeted therapies, are therefore needed. PD-L1 expression and microsatellite instability are predictive markers of the response to ICIs in solid tumors [88,89,90,91]. Nevertheless, in a series of forty pathologic specimens from patients with NECs of the cervix, PD-L1 was positive (considered as a composite proportion score ≥ 1) in only 8% of samples, while all were microsatellite-stable by IHC [92]. However, mutations in DNA damage response (DDR) genes, which can be commonly observed in GY-NECs, also induce the accumulation of DNA mutations, which cause high TMB [93], which is a predictive factor of the activity of ICIs in several malignancies [94,95]. Indeed, a cohort of the DART SWOG 1609 phase II study investigated the activity of the combination of ipilimumab and nivolumab in non-pancreatic neuroendocrine neoplasms [96]. The study enrolled three women with NENs of the cervix, of which one achieved a partial response. A few case reports of women with NENs of the cervix who received ICIs, both as single-agent nivolumab [95,97,98] or in combination with ipilimumab [99], as well as tislelizumab, a PD-1 inhibitor, combined with albumin-bound paclitaxel and anlotinib, a multi-kinase inhibitor with anti-angiogenic activity, suggested the potential utility of this class of drugs in this setting [100]. On the other hand, single-agent pembrolizumab, another PD-1 inhibitor, failed to improve survival in an unselected population enrolled in a small phase II trial [97,101]. Given the interest in investigating the efficacy of ICIs, especially for the novel PD-(L)1-CTLA-4 dual inhibitors, a phase II trial of cadonilimab, a bispecific anti-PD-1/CTLA-4 inhibitor, is ongoing for the second- and third-line setting in recurrent NECs of the cervix (NCT05063916) [8,102].

A deeper understanding of the molecular features of SCNECs of the cervix might help the implementation of targeted therapies. Medium-to-high PARP-1 expression by IHC has been observed in over 90% of NECs of cervix samples, suggesting a potential role for the use of PARP-1 inhibitors [92]. Furthermore, trametinib induced complete remission in a woman with a metastatic KRAS^G12D^ mutant SCNEC of the cervix lasting 8 months after treatment start [103].

Similarly, because NECs can harbor genomic alterations, such as NTRK or RET fusions, which can be successfully targeted by specific inhibitors, cervical NECs should undergo comprehensive next-generation sequencing to identify such alterations and provide potentially breakthrough therapeutic options [12,13,14].

### 4.3. NECs of the Endometrium

**Figure 3:**
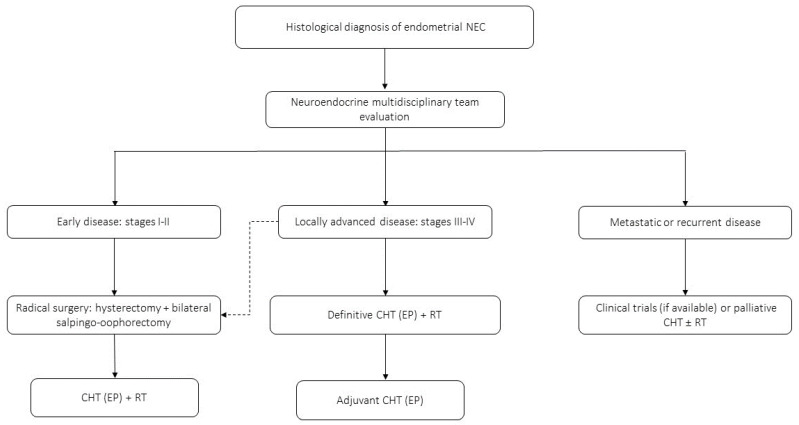
Proposed clinical management algorithm of endometrial NECs. For locally advanced disease, radical surgery could be considered in selected cases (dashed line).

Endometrial neuroendocrine carcinomas are extremely rare and represent 0.8% of all endometrial malignancies [104]. The most frequent clinical manifestations are unusual uterine bleeding or symptomatic metastases [105]. Endometrial NECs, which include small- and large-cell neoplasms, are highly aggressive cancers that entail more than double the risk of death compared to their non-neuroendocrine counterparts [104,106]. The average age of diagnosis is about 66 years [106] and in approximately 70% of cases, endometrial NECs are advanced tumors at diagnosis (FIGO stage III or IV) [68], with a reduced OS of 12 months when compared to 22 months in stages I–II [107]. Unlike endometrioid and undifferentiated carcinomas that present a focal positivity for neuroendocrine markers, the majority of endometrial NECs present a diffuse positivity for more than two neuroendocrine markers among neuron-specific enolase (NSE), CgA, synaptophysin, and CD56 [77]. The therapeutic approach for endometrial NECs is currently debated because of the lack of prospective data (Figure 3). For resectable tumors, surgery with total hysterectomy and bilateral salpingo-oophorectomy followed by postoperative radiotherapy and platinum + etoposide chemotherapy is the recommended approach. For advanced-stage tumors, platinum + etoposide chemotherapy is the recommended first-line treatment [77,108]. Of interest, an analysis of 170 patients with endometrial NETs from the SEER database, including SCNECs (N = 56), LCNECs (N = 60), and other NECs not elsewhere classified (N = 51), found that adding surgery to chemotherapy, with or without radiotherapy, may improve survival outcomes in patients with advanced disease [109], thus suggesting that surgery could be considered in select patients, when feasible.

### 4.4. NECs of the Ovary

**Figure 4:**
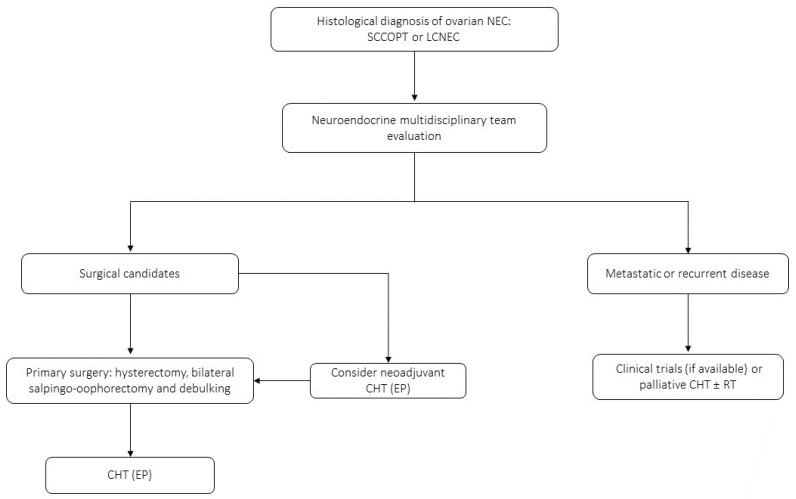
Proposed clinical management algorithm of ovarian NECs.

Among the ovarian NENs, NET is the most frequent subtype [68]. Ovarian NECs, which include SCNECs and LCNECs, are rare and aggressive tumors that represent less than 1–2% of all malignant ovarian neoplasms [79]. Among ovarian SCNECs, the following histological subtypes are recognized: the pulmonary type (SCCOPT), which is a proper NEN, and the hypercalcemic type (SCCOHT), which is an undifferentiated tumor with different grades of neuroendocrine immunoreactivity, not formally classified as a NEN and usually associated with symptomatic hypercalcemia [64,77]. SCCOPT generally affects women around 50 years old and occurs bilaterally in half of cases [77]. On the other hand, SCCOHT is usually diagnosed in younger women and is extremely aggressive, with a median OS between 5 and 14.5 months [110]. The ovarian LCNEC is the rarest variant among ovarian NENs (less than 10 cases have been reported in the literature so far) and is characterized by a highly aggressive behavior and a median survival of 10 months from diagnosis [111,112,113]. Given the rarity of these tumors, available data mainly derive from case reports and small case series, and thus, there are no dedicated recommendations or guidelines [77]. For small-cell ovarian carcinomas, the treatment involves surgical excision (hysterectomy, bilateral salpingo-oophorectomy and debulking), whenever possible, and cisplatin + etoposide chemotherapy as adjuvant treatment if following radical resection or as first-line treatment in metastatic cases (Figure 4) [74,79]. For advanced LCNECs, adopted regimens include chemotherapy with six cycles of cisplatin + etoposide or paclitaxel.

### 4.5. Other GY-NECs

Primary NECs of the vagina are extremely rare and aggressive malignancies, with less than 30 cases reported so far [114,115]. The average age at diagnosis is 59 years and the median OS for tumors with SCNEC histology approaches 11 months [115]. Due to the rarity of the tumor, there is no consensus regarding the optimal treatment. In the case of localized disease, chemoradiation with or without surgical resection (partial or total vaginectomy and lymphadenectomy) should be considered [79]. For advanced disease, palliative chemotherapy with cisplatin + etoposide is the preferred and most commonly used regimen [115,116]. NECs of the vulva are rare tumors that are classified among primary cutaneous neoplasms. According to the 2014 WHO classification of tumors of the female reproductive system, they are classified into Merkel cell carcinomas (MCCs), SCNECs, and LCNECs. Despite most of the cases being diagnosed as MCCs [63,79], in a retrospective pathology series of 16 cases, more than half were reclassified from MCCs to SCNECs by IHC and high-risk HPV testing [117]. Indeed, SCNECs and MCCs are histologically similar, with both tumor types staining for neuroendocrine markers, such as synaptophysin and CgA. Furthermore, around 30% of SCNECs express TTF-1, while 90% of MCCs stain positive for CK20 [118]. Both types of tumors show aggressive behavior with a median OS of 24 months, but therapeutic options are substantially different, e.g., with respect to indications for ICI treatment [119]. In women with early-stage disease, surgical resection followed by adjuvant chemoradiation is recommended for SCNECs and LCNECs. Primary chemoradiation is commonly proposed for unresectable, locally advanced cases, whereas chemotherapy with or without radiation of the primary tumor is advised in the case of metastatic disease [79,120].

## 5. NECs of the Genitourinary Tract

GU-NENs comprise 1% to 2% of all GU malignancies, and they account for 1.5% of all NECs [9].They are more common in women than men, with bladder as the most common site of tumor development [121,122]. Focusing only on male patients, neuroendocrine prostate cancer (NEPC) is the most frequent GU-NEC, while other locations include testicles, scrotum, penis, and penile urethra (mainly SCNECs) [1].

### 5.1. NECs of the Urinary Bladder

NENs of the urinary bladder can be classified according to the 2016 WHO/International Society of Urological Pathology(ISUP) classification in well-differentiated tumors, SCNECs, LCNECs, and paragangliomas [123]. Bladder SCNECs are rare entities, comprising only 0.5–1% of primary bladder malignancies. The pathogenesis and cell of origin of these tumors are uncertain, and they are usually found in association with either urothelial carcinoma, squamous-cell carcinoma, adenocarcinoma, or sarcomatoid carcinoma [124,125,126,127,128]. Bladder NEC typically affects older males, with a male-to-female ratio of 5:1 and a history of smoking. Hematuria associated with local irritation, pelvic pain, or urinary obstruction are the most common clinical symptoms at the time of diagnosis [129]. Macroscopically, SCNECs are not different from urothelial carcinomas and usually have a polypoid, solid, or ulcerative appearance with muscle or fat tissue invasion [7,130,131,132]. Microscopically, histological features of bladder SCNECs are similar to those of SCLC [7,132]. Tumor cells express both epithelial and neuroendocrine markers including CgA, synaptophysin, NSE, CD57, CD56, PGP9.5 and ‘dot-like’ cytokeratins. Mitotic count and Ki-67 index are essential for determining the correct tumor grade [133,134].

**Figure 5:**
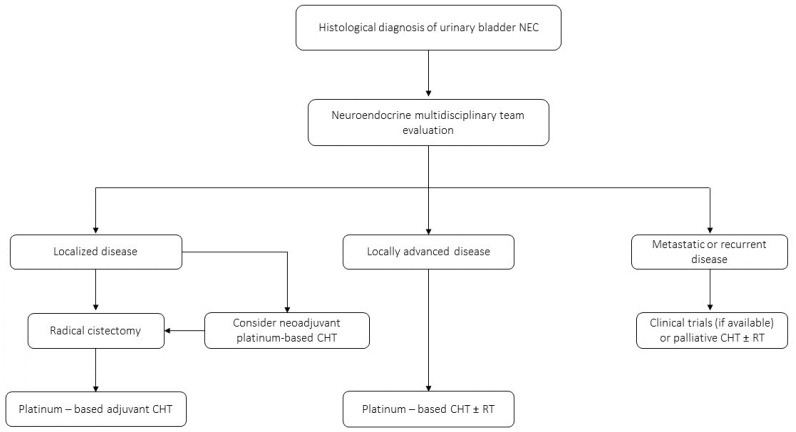
Proposed clinical management algorithm of urinary bladder NECs.

Bladder NECs have an aggressive clinical behavior, with median OS of approximately 20 months [135]. Therefore, considering the high risk of metastatic spread and the poor prognosis, a multimodal approach is recommended (Figure 5). Of note, considering its rarity and the absence of robust evidence coming from randomized clinical trials, the therapeutic management of this tumor subtype remains challenging. In the localized setting, transurethral resection of the bladder (TURB) alone is frequently ineffective in disease control. Prognostic factors at this stage are tumor size irrespective of the presence of nodal metastases (size cutoff: 45 mm), age (cutoff 72 years) and surgery performed in the context a node-positive disease [136]. Radical cystectomy preceded and/or followed by chemotherapy represent the gold standard in patients with stage I-III disease [124,137,138,139]. Retrospective studies have demonstrated a benefit with neoadjuvant platinum-based chemotherapy combined with radical cystectomy [140]. For locally advanced bladder NEC, platinum-based chemotherapy with or without radiation is the mainstay of treatment. However, due to the rarity of this malignancy, no prospective studies have been performed to establish the efficacy and duration of chemotherapy or the relative efficacy of platinum + etoposide versus other chemotherapeutic regimens. Finally, immunotherapy or chemo-immunotherapy could potentially represent a therapeutic option, however there are no data to conclusively support this strategy at present.

Bladder LCNEC is an extremely rare form of cancer with few cases reported in literature and seems to affect more commonly elderly patients [141,142]. Histologically, LCNEC is identical to large-cell carcinomas of the lungs, being characterized by large tumor cells with a polygonal shape, low nuclear to cytoplasmic ratio, coarse nuclear chromatin, prominent nucleoli and frequent mitotic figures [143,144]. Neuroendocrine markers mainly confirm the diagnosis, although CgA is expressed more frequently in bladder SCNEC than in LCNEC [145]. Bladder LCNEC is a tumor with high rate of local and distant recurrence, as well as poor prognosis (similar to SCNEC), thus requiring early diagnosis and aggressive combined treatment. Unfortunately, owing to the paucity of available data, mainly coming from case reports or case series, a standard algorithm for the diagnosis, treatment, and monitoring of this neoplasms is currently lacking. The most used therapy in the clinical practice is the platinum-based chemotherapy [7,146,147]. The potential role of immunotherapy or chemo-immunotherapy should be further investigated.

### 5.2. NECs of the Prostate

Primary NEPC is a rare disease, accounting for approximatively 1% of all prostatic malignancies at the time of initial diagnosis. More commonly, development of neuroendocrine differentiation emerges under androgen deprivation therapy later in the disease course of advanced prostate adenocarcinomas, and correlates with advanced disease, aggressive phenotype, refractoriness to standard therapies and poor outcomes. The incidence of NEPC is increasing due to more widespread use of highly potent antiandrogenic agents, such as abiraterone and enzalutamide. Overall, NEPC are diagnosed in approximately 20–25% of patients with advanced prostate cancer [148]. The biological mechanisms leading to NEPC development are still a matter of research. Growing evidence suggest that cellular plasticity and genetic reprogramming contribute to induction of androgen deprivation therapy (ADT) resistance and promotion of the transdifferentiation to neuroendocrine phenotype [149,150,151]. NEPC is characterized by several genomic and epigenomic alterations: loss of RB1 (70–90%) [152], loss of TP53 (56–67%) [153], overexpression and/or amplification of MYCN and AURKA (~40%) [154,155], ERG rearrangements (~50%) with one of the androgen-regulated genes (TMPRSS2, SLC45A3, and NDRG1) [156], codeletion of both MAP3K7 and CHD1 (10–20%) [157], upregulation of SOX2 [158] and/or PEG10 [159], upregulation of DNA methyltransferase and altered DNA methylation [153], upregulation of EZH2 [153].

NEPC present with rapidly progressive symptoms, predominantly visceral and lytic bone metastases, low serum PSA levels and rising levels of serum CgA and NSE. Paraneoplastic syndromes tend to develop more frequently in the context of NEPC than in patients with adenocarcinomas. Histologically, the differential diagnosis between NEPCs and prostate adenocarcinomas might be challenging. Neuroendocrine marker positivity and negative or weak prostate-specific antigen (PSA) staining can be used for differential diagnosis, as less than 20% of NEPCs retain PSA positivity. However, in a small proportion of cases (about 10%) even neuroendocrine marker (synaptophysin and CgA) expression can be negative. Moreover, approximatively 50% of NEPCs express TTF-1, limiting the utility of this marker for distinguishing between small-cell NEPCs and metastatic SCNECs from other organs [160]. The optimal management of NEPCs is not well established. Survival remains poor, ranging from 7 months to 2 years, and the limited therapeutic options available comprise platinum-based chemotherapy, which mirrors SCLC treatment as for other EP-NECs [161,162].

The evolving knowledge of biological mechanisms leading to neuroendocrine differentiation is contributing to the expansion of novel treatment modalities for this aggressive subtype of prostate cancer (Table 1 and Table 2). Alisertib inhibits N-Myc signaling and tumor growth by disrupting the interaction between N-Myc and its stabilizing factor Aurora-A kinase. Alisertib was investigated in a single-arm phase II trial that, although formally negative (median PFS: 2.2 months; median OS: 9.5 months), showed exceptional responses in a subset of patients with N-Myc and Aurora-A hyperactivity [163]. Similar to what is observed in prostate adenocarcinomas, ICI monotherapy showed poor efficacy in NEPCs. In a single-arm phase II study of the PD-L1 inhibitor avelumab an overall response rate of 7% was observed among patients with progressive microsatellite-stable (MSS) NEPCs [164].

Combination strategies with ICIs have been tested to enhance the effectiveness of immunotherapy in patients with NEPCs. In a small cohort of seven NEPC patients treated with platinum-based chemotherapy + pembrolizumab, the ORR was 43%, and the PFS and OS rate at 12 months were, respectively, 43% and 71% [165].

Talabostat (BXCL701) is an oral innate immune activator, and an inhibitor of dipeptidyl peptidases (DPPs) that triggers the inflammasome to alert and prime the immune cells, leading to the activation of adaptive immune cascade. The combination of BXCL701 and pembrolizumab has been evaluated in a phase IIa study in patients with platinum-pretreated NEPCs providing encouraging activity results (ORR: 20%; DCR: 48%; duration of response range: 1.8–9.8 months) [166]. Interestingly, all responders were MSS and/or low-TMB.

The Notch ligand Delta-like ligand 3 (DLL3) is aberrantly expressed on the cell surface of the majority of NEPCs. The DLL3 antibody drug conjugate rovalpituzumab tesirine (Rova-T) demonstrated preferential preclinical activity in NEPCs compared to prostate adenocarcinomas. These data supported the investigation of Rova-T as a potential therapeutic agent for NEPCs. The relative phase I/II study included a cohort of 101 patients with DLL3-positive NEC/NETs at multiple primary sites (NEPC: 21 patients) and demonstrated an apparently manageable toxicity profile in the presence of an ORR of 13% in pooled patients with NEC/NETs [167]. Unfortunately, the development of Rova-T was discontinued after two phase III studies failed to demonstrate a favorable risk–benefit balance of Rova-T in patients with SCLC.

Further approaches are still under investigation in NEPCs. Several trials are evaluating combinations of ICIs with chemotherapy (NCT04709276), targeted agents (NCT04848337), and the new generation of antiandrogen, apalutamide (NCT04926181).

Furthermore, DLL3 is also the target of two novel agents under evaluation in NEPCs: tarlatamab, a bispecific T-cell engager molecule that binds both DLL3 and CD3 (NCT04702737), and PT2017, a first-in-class bispecific antibody targeting DLL3 and CD47 (NCT05652686).

ESK981, a phase-I-cleared multi-target TKI, exhibited tumor growth inhibitory abilities by blocking the PIKfyve activity and disrupting autophagy. In preclinical models, better responses were observed in androgen-negative rather than in androgen-positive castration-resistant prostate cancers (CRPCs), suggesting a potential activity even in NEPCs [168]. The phase II multicohort study, including an NEPC cohort, is ongoing (NCT05988918). PRRT with Lu177-Dotatate could be an option in patients harboring somatostatin receptors and positive to 68-Ga-DOTATATE PET, which is under evaluation in a phase II trial (NCT05691465).

### 5.3. NECs of the Kidney

High-grade NECs of the kidney, which include SCNECs and LCNECs according to the 2022 WHO classification, are extremely rare and aggressive malignancies [169]. As neuroendocrine cells have never been described so far in the renal parenchyma, it is possible that kidney NECs might arise from multipotent stem cells with a neuroendocrine differentiation [7]. Due to the very low incidence of renal NECs, little is known about their clinical characteristics, and therapeutic strategies are yet very limited. A recent comprehensive systematic review, which also put together case reports and data from England’s National Cancer Registration and Analysis Service (NCRAS) registry, highlighted that renal NECs are diagnosed at a median age of 70 years (thus, in older individuals when compared with well-differentiated renal NETs), while inconsistent associations between sex and renal NENs have been found [170]. Furthermore, the primary tumors of renal NECs are larger in size than those of well-differentiated renal NETs, as about 20% of renal NECs have a tumor size greater than 10 cm, which is negatively associated with prognosis [170]. Indeed, 80% of renal NECs are metastatic at diagnosis, frequently spreading to the liver and bones, but also to the brain and lungs as reported in several case series [171]. Renal NEC does not seem to be associated with horseshoe kidney. Paraneoplastic syndromes, such as inappropriate antidiuretic hormone syndrome (SIADH), have been reported in a few cases of metastatic renal NECs [170,171]. Due to the rarity of this disease and the lack of prospective trials, no recommendations about the optimal therapeutic strategies, combined or sequential treatments, are available to date. There is some evidence that a platinum-based chemotherapy regimen can substantially impact the survival of these patients, since a median survival of 20 months was observed in patients with renal SCNECs receiving platinum-based chemotherapy, compared to 8 months in patients receiving other chemotherapy regimens without platinum [170]. Moreover, survival analyses were conducted in 63 patients with renal NENs reported in the NCRAS between 2012–2018, confirming the aggressiveness of this disease, as the 5-year survival of renal NECs was 38.4% [170]. Registry data have shown a significant correlation between stage and prognosis, even though the prognosis of these patients remains poor in any stage, with an OS of 3 months in patients with advanced renal SCNECs and an OS of 11 months in patients with localized renal SCNECs [171]. Renal LCNECs are even rarer and only a few case reports have been described so far, showing similar behavior and aggressiveness of SCNECs. Interestingly, a case of a 59-year old man with LCNECs of the kidney with cardiac metastasis was reported, confirming the cunning and unpredictable attitude of these diseases [172].

Comprehensive genomic profiling should be performed in order to detect potentially targetable drivers, always keeping in mind that the turnaround time of such analyses should not delay treatment start in a very aggressive disease that needs a prompt and efficient therapeutic strategy to cope with rapid tumor growth.

## 6. Conclusions

NECs have an extrapulmonary origin in less than 10% of cases. Overall, EP-NECs are characterized by a very low incidence, aggressive behavior, frequent presentation at later stages, high recurrence rate, and hence poor prognosis. The current strategy in the treatment of EP-NECs is extrapolated from SCLC, with platinum-based chemotherapy being the standard of care for EP-NECs of any primary sites. However, data about the efficacy of platinum-based combinations are limited and based only on case series or small retrospective studies. Referral to a dedicated neuroendocrine tumor center and discussion in a multidisciplinary team are essential for the management of such patients, especially as surgery is increasingly emerging, in select cases, as the primary treatment, capable of improving outcomes even in patients with locally advanced disease.

Expanding the therapeutic landscape of EP-NECs is of the utmost importance to improve patients’ prognosis. The current treatment options are indeed characterized by relatively short durations of response. Targeted therapies and ICIs appear as appealing agents for the treatment of EP-NECs given their tolerability profile, which compares favorably with that of standard chemotherapy, and the potential for long-term responses. Bench to bedside and back research focused on deciphering the mutational landscape of EP-NECs as well as understanding their immunological underpinnings will pave the way to innovative treatment strategies to be tested in the context of well-designed clinical trials modeled to reflect the rarity of these malignancies.

## Figures and Tables

**Table 1:** Summary of trials in prostate NEC.

	Study Design	NCT	Drug	Number of Patients	Primary Endpoint	Results	Study Completion
Avelumab [164]	Phase II single-arm	NCT03179410	Avelumab	15	ORR	ORR: 6.7% median rPFS 1.8 months (95% CI 1.6–3.6 months) Median OS: 7.4 months (85% CI 2.8–12.6 months)	2020
Pembrolizumab + Platin-based chemotherapy [165]	Phase I single-arm	NCT03582475	Pembrolizumab + platinum-based chemotherapy	14 (7 in cohort NEPC)	ORR, PFS, and OS	ORR: 43% PFS rate at 12 months: 43% OS rate at 12 months: 71%	2022
BXCL701 (Talabostat) + Pembrolizumab [166]	Phase IIa single-arm	NCT03910660	Talabostat + Pembrolizumab	34 (25 evaluable for response)	Composite response (either OR by RECIST 1.1 or PSA50 or CTC count conversion from ≥5/7.5 mL to <5/7.5 mL from baseline)	ORR 20% DCR 48%	Primary completion 2023 Study completion estimated 2025
Alisertib [163]	phase II single-arm	NCT01799278	Alisertib	60	6-month rPFS	mPFS:2.2 months (95% CI 2.0–2.6) mOS 9.5 months (95% CI 7.4–13.0)	2017
Rovalpituzumab tesirine (Rova-t)	Phase I/II	NCT02709889	Rovalpituzumab tesirine (Rova-t)	101 (NEC/NET cohort from multiple primary sites, 21 patiens with NEPC)	TEAEs	Grade 3/4 AEs: 54% [pleural effusion (5%), pericardial effusion (4%), and dyspnea (3%)] ORR overall population: 10% ORR NEC/NET: 13%	2019

AE: adverse event; CTCs: circulating tumor cells; DCR: disease control rate; mPFS: median progression-free survival; rPFS: radiological progression-free survival; mOS: median overall survival; NEC: neuroendocrine carcinoma; NEPC: neuroendocrine prostate carcinoma; NET: neuroendocrine tumor; ORR: overall response rate; TEAE: treatment-emergent adverse event.

**Table 2:** Summary of ongoing clinical trials in NEPC.

Name of Study and Population	Phase	Study Interventions	NCT Identifiers	Estimated Study Completion
Lu177-Dotatate in metastatic Prostate Cancer with Neuroendocrine Differentiation	II	Lutetium Lu 177-dotate (4 cycles)	NCT05691465	2024
BXCL701 and Pembrolizumab in Patients with mCRPC Small Cell Neuroendocrine Prostate Cancer Phenotype	IIb	BXCL701 +/− Pembrolizumab	NCT03910660	2025
PLANE-PC: Pembrolizumab and Lenvatinib in Advanced/Metastatic Neuroendocrine Prostate Cancer	II	Lenvatinib + Pembrolizumab	NCT04848337	2023
A Phase 1 Study of PT217 in Patients with Advanced Refractory Cancers Expressing DLL3 (NEPC, GEP-NET, SCLC, LCNEC)	I	PT217 (bispecific antibody against DDL3 and CD47)	NCT05652686	2025
Apalutamide Plus Cetrelimab in Patients with Treatment-Emergent Small Cell Neuroendocrine Prostate Cancer	II	Apalutamide + Cetrelimab (anti-PD1 antibody)	NCT04926181	2026
DeLLpro-300: A Study of Tarlatamab in Participants with Neuroendocrine Prostate Cancer	I	Tarlatamab (DLL3-rargeting BITE)	NCT04702737	2025
CHAMP: A Study of Chemoimmunotherapy for the Treatment of Men with Neuroendocrine or Aggressive Variant Metastatic Prostate Cancer	II	Nivolumab (w3), ipilimumab (w6), carboplatin (w3) and cabazitaxel (w3) × 10 cycles followed by maintenance nivolumab and ipilimumab (max 3 years)	NCT04709276	2027
Multicenter Trial of ESK981 in Patients with Select Solid Tumors (included cohort of NEPC)	II	ESK981	NCT05988918	2029

mCRPC: metastatic castration-resistant prostate cancer.

## Data Availability

Not applicable.

## References

[1] Garcia-Carbonero R., Sorbye H., Baudin E., Raymond E., Wiedenmann B., Niederle B., Sedlackova E., Toumpanakis C., Anlauf M., Cwikla J.B. (2016). ENETS consensus guidelines for high-grade gastroenteropancreatic neuroendocrine tumors and neuroendocrine carcinomas. Neuroendocrinology.

[2] Oronsky B., Ma P.C., Morgensztern D., Carter C.A. (2017). Nothing But NET: A Review of Neuroendocrine Tumors and Carcinomas. Neoplasia.

[3] Rindi G., Mete O., Uccella S., Basturk O., La Rosa S., Brosens L.A.A., Ezzat S., de Herder W.W., Klimstra D.S., Papotti M. (2022). Overview of the 2022 WHO Classification of Neuroendocrine Neoplasms.

[4] Classification of Tumours Editorial Board (2022). WHO Classification of Endocrine and Neuroendocrine Tumours.

[5] Rindi G., Wiedenmann B. (2020). Neuroendocrine neoplasia of the gastrointestinal tract revisited: Towards precision medicine. Nat. Rev. Endocrinol..

[6] Kaltsas G.A., Besser G.M., Grossman A.B. (2004). The diagnosis and medical management of advanced neuroendocrine tumors. Endocr. Rev..

[7] Shehabeldin A.N., Ro J.Y. (2019). Neuroendocrine tumors of genitourinary tract: Recent advances. Ann. Diagn. Pathol..

[8] Robinson M.D., Livesey D., Hubner R.A., Valle J.W., McNamara M.G. (2023). Future therapeutic strategies in the treatment of extrapulmonary neuroendocrine carcinoma: A review. Ther. Adv. Med. Oncol..

[9] Dasari A., Mehta K., Byers L.A., Sorbye H., Yao J.C. (2018). Comparative study of lung and extrapulmonary poorly differentiated neuroendocrine carcinomas: A SEER database analysis of 162,983 cases. Cancer.

[10] Uccella S., La Rosa S., Metovic J., Marchiori D., Scoazec J.Y., Volante M., Mete O., Papotti M. (2021). Genomics of High-Grade Neuroendocrine Neoplasms: Well-Differentiated Neuroendocrine Tumor with High-Grade Features (G3 NET) and Neuroendocrine Carcinomas (NEC) of Various Anatomic Sites. Endocr. Pathol..

[11] Rindi G., Klimstra D.S., Abedi-Ardekani B., Asa S.L., Bosman F.T., Brambilla E., Busam K.J., de Krijger R.R., Dietel M., El-Naggar A.K. (2018). A common classification framework for neuroendocrine neoplasms: An International Agency for Research on Cancer (IARC) and World Health Organization (WHO) expert consensus proposal. Mod. Pathol..

[12] Sigal D.S., Bhangoo M.S., Hermel J.A., Pavlick D.C., Frampton G., Miller V.A., Ross J.S., Ali S.M. (2018). Comprehensive genomic profiling identifies novel NTRK fusions in neuroendocrine tumors. Oncotarget.

[13] Subbiah V., Cassier P.A., Siena S., Garralda E., Paz-Ares L., Garrido P., Nadal E., Vuky J., Lopes G., Kalemkerian G.P. (2022). Pan-cancer efficacy of pralsetinib in patients with RET fusion–positive solid tumors from the phase 1/2 ARROW trial. Nat. Med..

[14] Doebele R.C., Drilon A., Paz-Ares L., Siena S., Shaw A.T., Farago A.F., Blakely C.M., Seto T., Cho B.C., Tosi D. (2020). Entrectinib in patients with advanced or metastatic NTRK fusion-positive solid tumours: Integrated analysis of three phase 1–2 trials. Lancet Oncol..

[15] Salama A.K.S., Li S., Macrae E.R., Park J.I., Mitchell E.P., Zwiebel J.A., Chen H.X., Gray R.J., McShane L.M., Rubinstein L.V. (2020). Dabrafenib and trametinib in patients with tumors with BRAFV600E mutations: Results of the NCI-MATCH trial subprotocol H. J. Clin. Oncol..

[16] Mortel C., Kvols L., O’Connell M. (1991). Treatment of neuroendocrine carcinomas with combined etoposide and cisplatin. Cancer.

[17] Ferro L.B., Wolf I., Peleg Hasson S., Golomb I., Osher E., Berlin A., Gutfeld O., Ospovat I., Soyfer V. (2021). Extrapulmonary Small Cell Cancer: A New Insight into a Rare Disease. Oncology.

[18] Krug S., Teupe F., Michl P., Gress T.M., Rinke A. (2019). Brain metastases in patients with neuroendocrine neoplasms: Risk factors and outcome. BMC Cancer.

[19] Dasari A., Shen C., Devabhaktuni A., Nighot R., Sorbye H. (2022). Survival According to Primary Tumor Location, Stage, and Treatment Patterns in Locoregional Gastroenteropancreatic High-grade Neuroendocrine Carcinomas. Oncologist.

[20] Alese O.B., Jiang R., Shaib W., Wu C., Akce M., Behera M., El-Rayes B.F. (2019). High-Grade Gastrointestinal Neuroendocrine Carcinoma Management and Outcomes: A National Cancer Database Study. Oncologist.

[21] Garcia-Carbonero R., Anton-Pascual B., Modrego A., Del Carmen Riesco-Martinez M., Lens-Pardo A., Carretero-Puche C., Rubio-Cuesta B., Soldevilla B. (2023). Advances in the Treatment of Gastroenteropancreatic Neuroendocrine Carcinomas: Are we Moving Forward?. Endocr. Rev..

[22] Pavel M., Öberg K., Falconi M., Krenning E.P., Sundin A., Perren A., Berruti A. (2020). Gastroenteropancreatic neuroendocrine neoplasms: ESMO Clinical Practice Guidelines for diagnosis, treatment and follow-up. Ann. Oncol..

[23] Deng H.Y., Ni P.Z., Wang Y.C., Wang W.P., Chen L.Q. (2016). Neuroendocrine carcinoma of the esophagus: Clinical characteristics and prognostic evaluation of 49 cases with surgical resection. J. Thorac. Dis..

[24] Eads J.R., Halfdanarson T.R., Asmis T., Belizzi A.M., Bergsland E.K., Dasari A., El-Haddad G., Frumovitz M., Meyer J., Mittra E. (2023). Expert Consensus Practice Recommendations of the North American Neuroendocrine Tumor Society for the management of high grade gastroenteropancreatic and gynecologic neuroendocrine neoplasms. Endocr. Relat. Cancer.

[25] Borbon L.C., Tran C.G., Sherman S.K., Ear P.H., Chandrasekharan C., Bellizzi A.M., Dillon J.S., O’Dorisio T.M., Howe J.R. (2022). ASO Visual Abstract: Is There a Role for Surgical Resection of Grade 3 Neuroendocrine Neoplasms?. Ann. Surg. Oncol..

[26] Fields A.C., Lu P., Vierra B.M., Hu F., Irani J., Bleday R., Goldberg J.E., Nash G.M. (2019). Carcinomas: The Role of Surgery and Chemotherapy. Ann. Surg. Oncol..

[27] Smith J.D., Reidy D.L., Goodman K.A., Shia J., Nash G.M. (2014). A retrospective review of 126 high-grade neuroendocrine carcinomas of the colon and rectum. Ann. Surg. Oncol..

[28] Holmager P., Langer S.W., Kjaer A., Ringholm L., Garbyal R.S., Pommergaard H.C., Hansen C.P., Federspiel B., Andreassen M., Knigge U. (2022). Surgery in Patients with Gastro-Entero-Pancreatic Neuroendocrine Carcinomas, Neuroendocrine Tumors G3 and High Grade Mixed Neuroendocrine-Non-Neuroendocrine Neoplasms. Curr. Treat. Options Oncol..

[29] Bertani E., Ravizza D., Milione M., Massironi S., Grana C.M., Zerini D., Piccioli A.N., Spinoglio G., Fazio N. (2018). Neuroendocrine neoplasms of rectum: A management update. Cancer Treat. Rev..

[30] Katada C., Komori S., Yoshida T., Kawakami S., Watanabe A., Ishido K., Azuma M., Wada T., Hosoda K., Yamashita K. (2020). A retrospective study of definitive chemoradiotherapy in patients with resectable small cell neuroendocrine carcinoma of the esophagus. Esophagus.

[31] Modrek A.S., Hsu H.C., Leichman C.G., Du K.L. (2015). Radiation therapy improves survival in rectal small cell cancer—Analysis of Surveillance Epidemiology and End Results (SEER) data. Radiat. Oncol..

[32] Brieau B., Lepere C., Walter T., Lecomte T., Guimbaud R., Manfredi S., Tougeron D., Desseigne F., Lourenco N., Afchain P. (2015). Radiochemotherapy versus surgery in nonmetastatic anorectal neuroendocrine carcinoma A multicenter study by the association des gastro-entérologues oncologues. Medicine.

[33] Strosberg J., El-Haddad G., Wolin E., Hendifar A., Yao J., Chasen B., Mittra E., Kunz P.L., Kulke M.H., Jacene H. (2017). Phase 3 Trial of 177 Lu-Dotatate for Midgut Neuroendocrine Tumors. N. Engl. J. Med..

[34] Konukiewitz B., Schlitter A.M., Jesinghaus M., Pfister D., Steiger K., Segler A., Agaimy A., Sipos B., Zamboni G., Weichert W. (2017). Somatostatin receptor expression related to TP53 and RB1 alterations in pancreatic and extrapancreatic neuroendocrine neoplasms with a Ki67-index above 20%. Mod. Pathol..

[35] Sorbye H., Welin S., Langer S.W., Vestermark L.W., Holt N., Osterlund P., Dueland S., Hofsli E., Guren M.G., Ohrling K. (2013). Predictive and prognostic factors for treatment and survival in 305 patients with advanced gastrointestinal neuroendocrine carcinoma (WHO G3): The NORDIC NEC study. Ann. Oncol..

[36] Nicolini S., Severi S., Ianniello A., Sansovini M., Ambrosetti A., Bongiovanni A., Scarpi E., Di Mauro F., Rossi A., Matteucci F. (2018). Investigation of receptor radionuclide therapy with 177Lu-DOTATATE in patients with GEP-NEN and a high Ki-67 proliferation index. Eur. J. Nucl. Med. Mol. Imaging.

[37] Carlsen E.A., Fazio N., Granberg D., Grozinsky-Glasberg S., Ahmadzadehfar H., Grana C.M., Zandee W.T., Cwikla J., Walter M.A., Oturai P.S. (2019). Peptide receptor radionuclide therapy in gastroenteropancreatic NEN G3: A multicenter cohort study. Endocr. Relat. Cancer.

[38] Filosso P.L., Guerrera F., Evangelista A., Galassi C., Welter S., Rendina E.A., Travis W., Lim E., Sarkaria I., Thomas P.A. (2017). Adjuvant chemotherapy for large-cell neuroendocrine lung carcinoma: Results from the European Society for Thoracic Surgeons Lung Neuroendocrine Tumours Retrospective Database. Eur. J. Cardio-Thorac. Surg..

[39] Merola E., Rinke A., Partelli S., Gress T.M., Andreasi V., Kollár A., Perren A., Christ E., Panzuto F., Pascher A. (2020). Surgery with Radical Intent: Is There an Indication for G3 Neuroendocrine Neoplasms?. Ann. Surg. Oncol..

[40] Mitry E., Rougier P. (2001). The treatment of undifferentiated neuroendocrine tumors. Crit. Rev. Oncol. Hematol..

[41] Zhang P., Li J., Li J., Zhang X., Zhou J., Wang X., Peng Z., Shen L., Lu M. (2020). Etoposide and cisplatin versus irinotecan and cisplatin as the first-line therapy for patients with advanced, poorly differentiated gastroenteropancreatic neuroendocrine carcinoma: A randomized phase 2 study. Cancer.

[42] Ardizzoni A., Tiseo M., Boni L. (2014). Validation of standard definition of sensitive versus refractory relapsed small cell lung cancer: A pooled analysis of topotecan second-line trials. Eur. J. Cancer.

[43] Hentic O., Hammel P., Couvelard A., Rebours V., Zappa M., Palazzo M., Maire F., Goujon G., Gillet A., Lévy P. (2012). FOLFIRI regimen: An effective second-line chemotherapy after failure of etoposide-platinum combination in patients with neuroendocrine carcinomas grade 3. Endocr. Relat. Cancer.

[44] Hadoux J., Malka D., Planchard D., Scoazec J.Y., Caramella C., Guigay J., Boige V., Leboulleux S., Burtin P., Berdelou A. (2015). Post-first-line FOLFOX chemotherapy for grade 3 neuroendocrine carcinoma. Endocr. Relat. Cancer.

[45] Welin S., Sorbye H., Sebjornsen S., Knappskog S., Busch C., Öberg K. (2011). Clinical effect of temozolomide-based chemotherapy in poorly differentiated endocrine carcinoma after progression on first-line chemotherapy. Cancer.

[46] Couronne T., Girot P., Hadoux J., Lecomte T., Durand A., Fine C., Vandevoorde K., Lombard-Bohas C., Walter T. (2020). Post first-line dacarbazine or temozolomide ineuroendocrine carcinoma. Endocr. Connect..

[47] Apostolidis L., Bergmann F., Jäger D., Winkler E.C. (2016). Efficacy of topotecan in pretreated metastatic poorly differentiated extrapulmonary neuroendocrine carcinoma. Cancer Med..

[48] McNamara M.G., Swain J., Craig Z., Sharma R., Faluyi O., Wadsley J., Morgan C., Wall L.R., Chau I., Reed N. (2023). NET-02: A randomised, non-comparative, phase II trial of nal-IRI/5-FU or docetaxel as second-line therapy in patients with progressive poorly differentiated extra-pulmonary neuroendocrine carcinoma. eClinicalMedicine.

[49] Walter T., Lievre A., Coriat R., Malka D., Elhajbi F., Di Fiore F., Hentic O., Smith D., Hautefeuille V., Roquin G. (2023). Bevacizumab plus FOLFIRI after failure of platinum–etoposide first-line chemotherapy in patients with advanced neuroendocrine carcinoma (PRODIGE 41-BEVANEC): A randomised, multicentre, non-comparative, open-label, phase 2 trial. Lancet Oncol..

[50] Bongiovanni A., Liverani C., Pusceddu S., Leo S., Di Meglio G., Tamberi S., Santini D., Gelsomino F., Pucci F., Berardi R. (2020). Randomised phase II trial of CAPTEM or FOLFIRI as SEcond-line therapy in NEuroendocrine CArcinomas and exploratory analysis of predictive role of PET/CT imaging and biological markers (SENECA trial): A study protocol. BMJ Open.

[51] Xing J., Ying H., Li J., Gao Y., Sun Z., Li J., Bai C., Cheng Y., Wu H. (2020). Immune Checkpoint Markers in Neuroendocrine Carcinoma of the Digestive System. Front. Oncol..

[52] Venizelos A., Elvebakken H., Perren A., Nikolaienko O., Deng W., Lothe I.M.B., Couvelard A., Hjortland G.O., Sundlöv A., Svensson J. (2022). The molecular characteristics of high-grade gastroenteropancreatic neuroendocrine neoplasms. Endocr. Relat. Cancer.

[53] Capdevila J., Hernando J., Teule A., Lopez C., Garcia-Carbonero R., Benavent M., Custodio A., Garcia-Alvarez A., Cubillo A., Alonso V. (2023). Durvalumab plus tremelimumab for the treatment of advanced neuroendocrine neoplasms of gastroenteropancreatic and lung origin. Nat. Commun..

[54] Girard N., Mazieres J., Otto J., Lena H., Lepage C., Egenod T., Smith D., Madelaine J., Gérinière L., El Hajbi F. (2021). LBA41 Nivolumab (nivo) ± ipilimumab (ipi) in pre-treated patients with advanced, refractory pulmonary or gastroenteropancreatic poorly differentiated neuroendocrine tumors (NECs) (GCO-001 NIPINEC). Ann. Oncol..

[55] Horn L., Mansfield A.S., Szczęsna A., Havel L., Krzakowski M., Hochmair M.J., Huemer F., Losonczy G., Johnson M.L., Nishio M. (2018). First-Line Atezolizumab plus Chemotherapy in Extensive-Stage Small-Cell Lung Cancer. N. Engl. J. Med..

[56] Riesco Martinez M.C., Capdevila Castillon J., Alonso V., Jimenez-Fonseca P., Teule A., Grande E., Sevilla I., Benavent M., Alonso-Gordoa T., Custodio A. (2022). 496MO Final overall survival results from the NICE-NEC trial (GETNE-T1913): A phase II study of nivolumab and platinum-doublet chemotherapy (CT) in untreated advanced G3 neuroendocrine neoplasms (NENs) of gastroenteropancreatic (GEP) or unknown (UK) origi. Ann. Oncol..

[57] Hong D.S., Fakih M.G., Strickler J.H., Desai J., Durm G.A., Shapiro G.I., Falchook G.S., Price T.J., Sacher A., Denlinger C.S. (2020). KRAS G12C Inhibition with Sotorasib in Advanced Solid Tumors. N. Engl. J. Med..

[58] Bekaii-Saab T.S., Yaeger R., Spira A.I., Pelster M.S., Sabari J.K., Hafez N., Barve M., Velastegui K., Yan X., Shetty A. (2023). Adagrasib in Advanced Solid Tumors Harboring a KRAS G12C Mutation. J. Clin. Oncol..

[59] Subbiah V., Puzanov I., Blay J.Y., Chau I., Lockhart A.C., Raje N.S., Wolf J., Baselga J., Meric-Bernstam F., Roszik J. (2020). Pan-cancer efficacy of vemurafenib in brafv600-mutant non-melanoma cancers. Cancer Discov..

[60] Sennino B., Ishiguro-oonuma T., Wei Y., Naylor R.M., Williamson C.W. (2012). Suppression of Tumor Invasion and Metastasis by Concurrent Inhibition of c-Met and VEGF Signaling in Pancreatic Neuroendocrine Tumors. Cancer Discov..

[61] Pellat A., Dreyer C., Couffignal C., Walter T., Lombard-Bohas C., Niccoli P., Seitz J.F., Hentic O., André T., Coriat R. (2018). Clinical and Biomarker Evaluations of Sunitinib in Patients with Grade 3 Digestive Neuroendocrine Neoplasms. Neuroendocrinology.

[62] Alifieris C.E., Griniatsos J., Delis S.G., Nikolaou M., Avgoustou C., Panagiotidis M.I., Souferi-Chronopoulou E., Trafalis D.T. (2020). Capecitabine, Oxaliplatin, Irinotecan, and Bevacizumab Combination Followed by Pazopanib plus Capecitabine Maintenance for High-Grade Gastrointestinal Neuroendocrine Carcinomas. Am. J. Clin. Oncol. Cancer Clin. Trials.

[63] Patibandla J.R., Fehniger J.E., Levine D.A., Jelinic P. (2018). Small cell cancers of the female genital tract: Molecular and clinical aspects. Gynecol. Oncol..

[64] Georgescu T.A., Bohiltea R.E., Munteanu O., Furtunescu F., Lisievici A.C., Grigoriu C., Gherghiceanu F., Vlădăreanu E.M., Berceanu C., Ducu I. (2021). Emerging therapeutic concepts and latest diagnostic advancements regarding neuroendocrine tumors of the gynecologic tract. Medicina.

[65] Bermúdez A., Vighi S., García A., Sardi J. (2001). Neuroendocrine cervical carcinoma: A diagnostic and therapeutic challenge. Gynecol. Oncol..

[66] Zou Q., Zhang L., Cheng Z., Guo X., Cao D. (2021). INSM1 Is Less Sensitive but More Specific Than Synaptophysin in Gynecologic High-grade Neuroendocrine Carcinomas. Am. J. Surg. Pathol..

[67] Crane E.K., Ramos P., Farley J.H., Naumann R.W., Tait D.L., Higgins R.V., Brown J. (2020). Molecular profiling in a large cohort of gynecologic neuroendocrine tumors. Gynecol. Oncol..

[68] Caruso G., Sassu C.M., Tomao F., Di Donato V., Perniola G., Fischetti M., Benedetti Panici P., Palaia I. (2021). The puzzle of gynecologic neuroendocrine carcinomas: State of the art and future directions. Crit. Rev. Oncol. Hematol..

[69] Gadducci A., Carinelli S., Aletti G. (2017). Neuroendrocrine tumors of the uterine cervix: A therapeutic challenge for gynecologic oncologists. Gynecol. Oncol..

[70] Castle P.E., Pierz A., Stoler M.H. (2018). A systematic review and meta-analysis on the attribution of human papillomavirus (HPV) in neuroendocrine cancers of the cervix. Gynecol. Oncol..

[71] Alejo M., Alemany L., Clavero O., Quiros B., Vighi S., Seoud M., Cheng-Yang C., Garland S.M., Juanpere N., Lloreta J. (2018). Contribution of Human papillomavirus in neuroendocrine tumors from a series of 10,575 invasive cervical cancer cases. Papillomavirus Res..

[72] Frumovitz M., Burzawa J.K., Byers L.A., Lyons Y.A., Ramalingam P., Coleman R.L., Brown J. (2016). Sequencing of mutational hotspots in cancer-related genes in small cell neuroendocrine cervical cancer. Gynecol. Oncol..

[73] Xing D., Zheng G., Schoolmeester J.K., Li Z., Pallavajjala A., Haley L., Conner M.G., Vang R., Hung C.F., Wu T.C. (2018). Next-generation Sequencing Reveals Recurrent Somatic Mutations in Small Cell Neuroendocrine Carcinoma of the Uterine Cervix. Am. J. Surg. Pathol..

[74] Winer I., Kim C., Gehrig P. (2021). Neuroendocrine tumors of the gynecologic tract update. Gynecol. Oncol..

[75] Bhatla N., Aoki D., Sharma D.N., Sankaranarayanan R. (2018). Cancer of the cervix uteri. Int. J. Gynecol. Obstet..

[76] McCusker M.E., Coté T.R., Clegg L.X., Tavassoli F.J. (2003). Endocrine tumors of the uterine cervix: Incidence, demographics, and survival with comparison to squamous cell carcinoma. Gynecol. Oncol..

[77] Virarkar M., Vulasala S.S., Morani A.C., Waters R., Gopireddy D.R., Kumar S., Bhosale P., Lall C. (2022). Neuroendocrine Neoplasms of the Gynecologic Tract. Cancers.

[78] Tempfer C.B., Tischoff I., Dogan A., Hilal Z., Schultheis B., Kern P., Rezniczek G.A. (2018). Neuroendocrine carcinoma of the cervix: A systematic review of the literature. BMC Cancer.

[79] Gardner G.J., Reidy-Lagunes D., Gehrig P.A. (2011). Neuroendocrine tumors of the gynecologic tract: A Society of Gynecologic Oncology (SGO) clinical document. Gynecol. Oncol..

[80] Chu T., Meng Y., Wu P., Li Z., Wen H., Ren F., Zou D., Lu H., Wu L., Zhou S. (2023). The prognosis of patients with small cell carcinoma of the cervix: A retrospective study of the SEER database and a Chinese multicentre registry. Lancet Oncol..

[81] Kim C., Salvo G., Ishikawa M., Chen T.C., Jhingran A., Bhosale P., Ramalingam P., Frumovitz M. (2023). The role of postoperative radiation after radical hysterectomy for women with early-stage neuroendocrine carcinoma of the cervix: A meta-analysis. Gynecol. Oncol..

[82] Boruta D.M., Schorge J.O., Duska L.A., Crum C.P., Castrillon D.H., Sheets E.E. (2001). Multimodality therapy in early-stage neuroendocrine carcinoma of the uterine cervix. Gynecol. Oncol..

[83] Hoskins P.J., Swenerton K.D., Pike J.A., Lim P., Aquino-Parsons C., Wong F., Lee N. (2003). Small-cell carcinoma of the cervix: Fourteen years of experience at a single institution using a combined-modality regimen of involved-field irradiation and platinum-based combination chemotherapy. J. Clin. Oncol..

[84] Salvo G., Martin A.G., Gonzales N.R., Frumovitz M. (2019). Updates and management algorithm for neuroendocrine tumors of the uterine cervix. Int. J. Gynecol. Cancer.

[85] Frumovitz M., Munsell M.F., Burzawa J.K., Byers L.A., Ramalingam P., Brown J., Coleman R.L. (2017). Combination therapy with topotecan, paclitaxel, and bevacizumab improves progression-free survival in recurrent small cell neuroendocrine carcinoma of the cervix. Gynecol. Oncol..

[86] Tewari K.S., Sill M.W., Long H.J., Penson R.T., Huang H., Ramondetta L.M., Landrum L.M., Oaknin A., Reid T.J., Leitao M.M. (2017). 1Final overall survival of the phase III randomised trial of chemotherapy with and without bevacizumab for advanced cervical cancer: An NRG oncology/gynecologic oncology group study. Obstet. Gynecol. Surv..

[87] Tangjitgamol S., Ramirez P.T., Sun C.C., See H.T., Jhingran A., Kavanagh J.J., Deavers M.T. (2005). Expression of HER-2/neu, epidermal growth factor receptor, vascular endothelial growth factor, cyclooxygenase-2, estrogen receptor, and progesterone receptor in small cell and large cell neuroendocrine carcinoma of the uterine cervix: A clinicopathologic. Int. J. Gynecol. Cancer.

[88] Garon E.B., Rizvi N.A., Hui R., Leighl N., Balmanoukian A.S., Eder J.P., Patnaik A., Aggarwal C., Gubens M., Horn L. (2015). Pembrolizumab for the Treatment of Non–Small-Cell Lung Cancer. N. Engl. J. Med..

[89] Paver E.C., Cooper W.A., Colebatch A.J., Ferguson P.M., Hill S.K., Lum T., Shin J.S., O’Toole S., Anderson L., Scolyer R.A. (2021). Programmed death ligand-1 (PD-L1) as a predictive marker for immunotherapy in solid tumours: A guide to immunohistochemistry implementation and interpretation. Pathology.

[90] Aguilar E.J., Ricciuti B., Gainor J.F., Kehl K.L., Kravets S., Dahlberg S., Nishino M., Sholl L.M., Adeni A., Subegdjo S. (2019). Outcomes to first-line pembrolizumab in patients with non-small-cell lung cancer and very high PD-L1 expression. Ann. Oncol..

[91] Le D.T., Durham J.N., Smith K.N., Wang H., Bjarne R., Aulakh L.K., Lu S., Kemberling H., Wilt C., Brandon S. (2017). Mismatch repair deficiency predicts response of solid tumors to PD-1 blockade. Science.

[92] Carroll M.R., Ramalingam P., Salvo G., Fujimoto J., Solis Soto L.M., Phoolcharoen N., Hillman R.T., Cardnell R., Byers L., Frumovitz M. (2020). Evaluation of PARP and PDL-1 as potential therapeutic targets for women with high-grade neuroendocrine carcinomas of the cervix. Int. J. Gynecol. Cancer.

[93] Mahdi H., Joehlin-Price A., Elishaev E., Dowlati A., Abbas A. (2021). Genomic analyses of high-grade neuroendocrine gynecological malignancies reveal a unique mutational landscape and therapeutic vulnerabilities. Mol. Oncol..

[94] Wu H.-X., Wang Z.-X., Zhao Q., Chen D.-L., He M.-M., Yang L.-P., Wang Y.-N., Jin Y., Ren C., Luo H.-Y. (2019). Tumor mutational and indel burden: A systematic pan-cancer evaluation as prognostic biomarkers. Ann. Transl. Med..

[95] Ricciuti B., Wang X., Alessi J.V., Rizvi H., Mahadevan N.R., Li Y.Y., Polio A., Lindsay J., Umeton R., Sinha R. (2022). Association of High Tumor Mutation Burden in Non-Small Cell Lung Cancers with Increased Immune Infiltration and Improved Clinical Outcomes of PD-L1 Blockade Across PD-L1 Expression Levels. JAMA Oncol..

[96] Patel S.P., Othus M., Chae Y.K., Giles F.J., Hansel D.E., Singh P.P., Fontaine A., Shah M.H., Kasi A., Baghdadi T.A. (2020). A Phase II Basket Trial of Dual Anti–CTLA-4 and Anti–PD-1 Blockade in Rare Tumors (DART SWOG 1609) in Patients with Nonpancreatic Neuroendocrine Tumors. Clin. Cancer Res..

[97] Sharabi A., Kim S.S., Kato S., Sanders P.D., Patel S.P., Sanghvi P., Weihe E., Kurzrock R. (2017). Exceptional Response to Nivolumab and Stereotactic Body Radiation Therapy (SBRT) in Neuroendocrine Cervical Carcinoma with High Tumor Mutational Burden: Management Considerations from the Center for Personalized Cancer Therapy at UC San Diego Moores Cance. Oncologist.

[98] Paraghamian S.E., Longoria T.C., Eskander R.N. (2017). Metastatic small cell neuroendocrine carcinoma of the cervix treated with the PD-1 inhibitor, nivolumab: A case report. Gynecol. Oncol. Res. Pract..

[99] Paterniti T.A., Dorr K., Ullah A., White J., Williams H., Ghamande S. (2021). Complete Response to Combination Nivolumab and Ipilimumab in Recurrent Neuroendocrine Carcinoma of the Cervix. Obstet. Gynecol..

[100] Ji X., Sui L., Song K., Lv T., Zhao H., Yao Q. (2021). PD-L1, PARP1, and MMRs as potential therapeutic biomarkers for neuroendocrine cervical cancer. Cancer Med..

[101] Frumovitz M., Westin S.N., Salvo G., Zarifa A., Xu M., Yap T.A., Rodon A.J., Karp D.D., Abonofal A., Jazaeri A.A. (2020). Phase II study of pembrolizumab efficacy and safety in women with recurrent small cell neuroendocrine carcinoma of the lower genital tract. Gynecol. Oncol..

[102] Frumovitz M., Chisholm G.B., Jhingran A., Ramalingam P., Flores-Legarreta A., Bhosale P., Gonzales N.R., Hillman R.T., Salvo G. (2023). Combination therapy with topotecan, paclitaxel, and bevacizumab improves progression-free survival in patients with recurrent high-grade neuroendocrine cervical cancer: A Neuroendocrine Cervical Tumor Registry (NeCTuR) study. Am. J. Obstet. Gynecol..

[103] Lyons Y.A., Frumovitz M., Soliman P.T. (2014). Response to MEK inhibitor in small cell neuroendocrine carcinoma of the cervix with a KRAS mutation. Gynecol. Oncol. Rep..

[104] Schlechtweg K., Chen L., St. Clair C.M., Tergas A.I., Khoury-Collado F., Hou J.Y., Melamed A., Neugut A.I., Hershman D.L., Wright J.D. (2019). Neuroendocrine carcinoma of the endometrium: Disease course, treatment, and outcomes. Gynecol. Oncol..

[105] Akgor U., Kuru O., Sakinci M., Boyraz G., Sen S., Cakır I., Turan T., Gokcu M., Gultekin M., Sayhan S. (2021). Neuroendocrine carcinoma of the endometrium: A very rare gynecologic malignancy. J. Gynecol. Obstet. Hum. Reprod..

[106] Zhang Z., Wang J., Wu X., Liu Y., Xi X. (2022). Clinical characteristic and prognostic factors in high-grade endometrial neuroendocrine carcinoma. J. Obstet. Gynaecol. Res..

[107] Lopes Dias J., Cunha T.M., Gomes F.V., Callé C., Félix A. (2015). Neuroendocrine tumours of the female genital tract: A case-based imaging review with pathological correlation. Insights Imaging.

[108] Pocrnich C.E., Ramalingam P., Euscher E.D., Malpica A. (2016). Neuroendocrine carcinoma of the endometrium: A clinicopathologic study of 25 cases. Am. J. Surg. Pathol..

[109] Zhang J., Pang L. (2022). Primary Neuroendocrine Tumors of the Endometrium: Management and Outcomes. Front. Oncol..

[110] Witkowski L., Goudie C., Ramos P., Boshari T., Brunet J.S., Karnezis A.N., Longy M., Knost J.A., Saloustros E., McCluggage W.G. (2016). The influence of clinical and genetic factors on patient outcome in small cell carcinoma of the ovary, hypercalcemic type. Gynecol. Oncol..

[111] Gupta P., Bagga R., Rai B., Srinivasan R. (2021). Primary pure large cell neuroendocrine carcinoma of the ovary: Histopathologic and immunohistochemical analysis with review of the literature. Int. J. Clin. Exp. Pathol..

[112] Pang L., Guo Z. (2021). Primary neuroendocrine tumors of the ovary: Management and outcomes. Cancer Med..

[113] Jang A., Newell M.J., Phaeton R., Kesterson P.J. (2016). Large cell neuroendocrine carcinoma (LCNEC) of the ovary: A case report and review of the literature. Integr. Cancer Sci. Ther..

[114] Bhalodia J.N., Kapapura D.V., Parekh M.N. (2011). Primary Small Cell Neuroendocrine Carcinoma of Vagina: A Rare Case Report. Patholog. Res. Int..

[115] Bing Z., Levine L., Lucci J.A., Hatch S.S., Eltorky M.A. (2004). Primary small cell neuroendocrine carcinoma of the vagina: A clinicopathologic study. Arch. Pathol. Lab. Med..

[116] Oliveira R., Bócoli M.C., Saldanha J.C., Murta E.F.C., Nomelini R.S. (2013). Primary Small Cell Carcinoma of the Vagina. Case Rep. Obstet. Gynecol..

[117] Chen P.P., Ramalingam P., Alvarado-Cabrero I., Euscher E.D., Nagarajan P., Lawson B.C., Malpica A. (2021). High-grade Neuroendocrine Carcinomas of the Vulva: A Clinicopathologic Study of 16 Cases. Am. J. Surg. Pathol..

[118] Bobos M., Hytiroglou P., Kostopoulos I., Karkavelas G., Papadimitriou C.S. (2006). Immunohistochemical distinction between Merkel cell carcinoma and small cell carcinoma of the lung. Am. J. Dermatopathol..

[119] Maggio I., Manuzzi L., Lamberti G., Ricci A.D., Tober N., Campana D. (2020). Landscape and future perspectives of immunotherapy in neuroendocrine neoplasia. Cancers.

[120] Eckert F., Fehm T., Bamberg M., Müller A.C. (2010). Small cell carcinoma of vulva: Curative multimodal treatment in face of resistance to initial standard chemotherapy. Strahlenther. Onkol..

[121] Virarkar M., Vulasala S.S., Gopireddy D., Morani A.C., Daoud T., Waters R., Bhosale P. (2022). Neuroendocrine Neoplasms of the Female Genitourinary Tract: A Comprehensive Overview. Cancers.

[122] Le B.K., McGarrah P., Paciorek A., Mohamed A., Apolo A.B., Chan D.L., Reidy-Lagunes D., Hauser H., Rivero J.D., Whitman J. (2023). Urinary Neuroendocrine Neoplasms Treated in the “Modern Era”: A Multicenter Retrospective Review. Clin. Genitourin. Cancer.

[123] Humphrey P.A., Moch H., Cubilla A.L., Ulbright T.M., Reuter V.E. (2016). The 2016 WHO Classification of Tumours of the Urinary System and Male Genital Organs—Part B: Prostate and Bladder Tumours. Eur. Urol..

[124] Blomjous C.E., Vos W., De Voogt H.J., Van der Valk P., Meijer C.J. (1989). Small cell carcinoma of the urinary bladder. A clinicopathologic, morphometric, immunohistochemical, and ultrastructural study of 18 cases. Cancer.

[125] Erdem G.U., Özdemir N.Y., Demirci N.S., Şahin S., Bozkaya Y., Zengin N. (2016). Small cell carcinoma of the urinary bladder: Changing trends in the current literature. Curr. Med. Res. Opin..

[126] Ploeg M., Aben K.K., Hulsbergen-van de Kaa C.A., Schoenberg M.P., Witjes J.A., Kiemeney L.A. (2010). Clinical Epidemiology of Nonurothelial Bladder Cancer: Analysis of The Netherlands Cancer Registry. J. Urol..

[127] Pompas-Veganzones N., Gonzalez-Peramato P., Sanchez-Carbayo M. (2014). The neuroendocrine component in bladder tumors. Curr. Med. Chem..

[128] Grignon D.J., Ro J.Y., Ayala A.G., Shum D.T., Ordóñez N.G., Logothetis C.J., Johnson D.E., Mackay B. (1992). Small cell carcinoma of the urinary bladder. A clinicopathologic analysis of 22 cases. Cancer.

[129] Bertaccini A., Marchiori D., Cricca A., Garofalo M., Giovannini C., Manferrari F., Gerace T.G., Pernetti R., Martorana G. (2008). Neuroendocrine carcinoma of the urinary bladder: Case report and review of the literature. Anticancer Res..

[130] Cheng L., Pan C.X., Yang X.J., Lopez-Beltran A., MacLennan G.T., Lin H., Kuzel T.M., Papavero V., Tretiakova M., Nigro K. (2004). Small cell carcinoma of the urinary bladder: A clinicopathologic analysis of 64 patients. Cancer.

[131] Sjödahl G. (2018). Molecular subtype profiling of urothelial carcinoma using a subtype-specific immunohistochemistry panel. Methods Mol. Biol..

[132] Wang X., MacLennan G.T., Lopez-Beltran A., Cheng L. (2007). Small cell carcinoma of the urinary bladder—Histogenesis, genetics, diagnosis, biomarkers, treatment, and prognosis. Appl. Immunohistochem. Mol. Morphol..

[133] Ghervan L., Zaharie A., Ene B., Elec F.I. (2017). Small-cell carcinoma of the urinary bladder: Where do we stand?. Clujul Med..

[134] Zhou H.H., Liu L.Y., Yu G.H., Qu G.M., Gong P.Y., Yu X., Yang P. (2017). Analysis of clinicopathological features and prognostic factors in 39 cases of bladder neuroendocrine carcinoma. Anticancer Res..

[135] Naturale R.T., MacLennan G.T. (2006). Small Cell Carcinoma of the Bladder. J. Urol..

[136] Mollica V., Massari F., Andrini E., Rosellini M., Marchetti A., Nuvola G., Tassinari E., Lamberti G., Campana D. (2022). Prognostic Factors of Survival for High-Grade Neuroendocrine Neoplasia of the Bladder: A SEER Database Analysis. Curr. Oncol..

[137] Smith J., Reidy-Lagunes D. (2013). The management of extrapulmonary poorly differentiated (high-grade) neuroendocrine carcinomas. Semin. Oncol..

[138] Vetterlein M.W., Wankowicz S.A.M., Seisen T., Lander R., Löppenberg B., Chun F.K.H., Menon M., Sun M., Barletta J.A., Choueiri T.K. (2017). Neoadjuvant chemotherapy prior to radical cystectomy for muscle-invasive bladder cancer with variant histology. Cancer.

[139] Lamberti G., Brizzi M.P., Pusceddu S., Gelsomino F., Di Meglio G., Massari F., Badalamenti G., Riccardi F., Ibrahim T., Ciccarese C. (2020). Perioperative Chemotherapy in Poorly Differentiated Neuroendocrine Neoplasia of the Bladder: A Multicenter Analysis. J. Clin. Med..

[140] Jung K., Ghatalia P., Litwin S., Horwitz E.M., Uzzo R.G., Greenberg R.E., Viterbo R., Geynisman D.M., Kutikov A., Plimack E.R. (2017). Small-Cell Carcinoma of the Bladder: 20-Year Single-Institution Retrospective Review. Clin. Genitourin. Cancer.

[141] Mondal K., Mandal R. (2017). A carcinoid tumor in the urinary bladder with uncommon clinicopathological presentation. Iran. J. Pathol..

[142] Coelho H.M.P., Pereira B.A.G.J., Caetano P.A.S.T. (2014). Large cell neuroendocrine carcinoma of the urinary bladder: Case report and review. Curr. Urol..

[143] Akdeniz E., Bakirtas M., Bolat M.S., Akdeniz S., Özer I. (2018). Pure large cell neuroendocrine carcinoma of the bladder without urological symptoms. Pan Afr. Med. J..

[144] Gupta S., Thompson R.H., Boorjian S.A., Thapa P., Hernandez L.P.H., Jimenez R.E., Costello B.A., Frank I., Cheville J.C. (2015). High grade neuroendocrine carcinoma of the urinary bladder treated by radical cystectomy: A series of small cell, mixed neuroendocrine and large cell neuroendocrine carcinoma. Pathology.

[145] Radović N., Turner R., Bacalja J. (2015). Primary “Pure” Large Cell Neuroendocrine Carcinoma of the Urinary Bladder: A Case Report and Review of the Literature. Clin. Genitourin. Cancer.

[146] Pósfai B., Kuthi L., Varga L., Laczo I., Révész J., Kránicz R., Maráz A. (2018). The colorful palette of neuroendocrine neoplasms in the genitourinary tract. Anticancer Res..

[147] Mazzucchelli R., Morichetti D., Lopez-Beltran A., Cheng L., Scarpelli M., Kirkali Z., Montironi R. (2009). Neuroendocrine tumours of the urinary system and male genital organs: Clinical significance. BJU Int..

[148] Aggarwal R., Huang J., Alumkal J.J., Zhang L., Feng F.Y., Thomas G.V., Weinstein A.S., Friedl V., Zhang C., Witte O.N. (2018). Clinical and genomic characterization of treatment-emergent small-cell neuroendocrine prostate cancer: A multi-institutional prospective study. J. Clin. Oncol..

[149] Zou M., Toivanen R., Mitrofanova A., Floch N., Hayati S., Sun Y., Le Magnen C., Chester D., Mostaghel E.A., Califano A. (2017). Transdifferentiation as a mechanism of treatment resistance in a mouse model of castration-resistant prostate cancer. Cancer Discov..

[150] Guo C.C., Dancer J.Y., Wang Y., Aparicio A., Navone N.M., Troncoso P., Czerniak B.A. (2011). TMPRSS2-ERG gene fusion in small cell carcinoma of the prostate. Hum. Pathol..

[151] Mounir Z., Lin F., Lin V.G., Korn J.M., Yu Y., Valdez R., Aina O.H., Buchwalter G., Jaffe A.B., Korpal M. (2015). TMPRSS2:ERG blocks neuroendocrine and luminal cell differentiation to maintain prostate cancer proliferation. Oncogene.

[152] Tan H.L., Sood A., Rahimi H.A., Wang W., Gupta N., Hicks J., Mosier S., Gocke C.D., Epstein J.I., Netto G.J. (2014). Rb loss is characteristic of prostatic small cell neuroendocrine carcinoma. Clin. Cancer Res..

[153] Beltran H., Prandi D., Mosquera J.M., Benelli M., Puca L., Cyrta J., Marotz C., Giannopoulou E., Chakravarthi B.V.S.K., Varambally S. (2016). Divergent clonal evolution of castration-resistant neuroendocrine prostate cancer. Nat. Med..

[154] Beltran H., Rickman D.S., Park K., Chae S.S., Sboner A., MacDonald T.Y., Wang Y., Sheikh K.L., Terry S., Tagawa S.T. (2011). Molecular characterization of neuroendocrine prostate cancer and identification of new drug targets. Cancer Discov..

[155] Lee J.K., Phillips J.W., Smith B.A., Park J.W., Stoyanova T., McCaffrey E.F., Baertsch R., Sokolov A., Meyerowitz J.G., Mathis C. (2016). N-Myc Drives Neuroendocrine Prostate Cancer Initiated from Human Prostate Epithelial Cells. Cancer Cell.

[156] Lotan T.L., Gupta N.S., Wang W., Toubaji A., Haffner M.C., Chaux A., Hicks J.L., Meeker A.K., Bieberich C.J., De Marzo A.M. (2011). ERG gene rearrangements are common in prostatic small cell carcinomas. Mod. Pathol..

[157] Rodrigues L.U., Rider L., Nieto C., Romero L., Karimpour-Fard A., Loda M., Lucia M.S., Wu M., Shi L., Cimic A. (2015). Coordinate loss of MAP3K7 and CHD1 promotes aggressive prostate cancer. Cancer Res..

[158] Mu P., Zhang Z., Benelli M., Karthaus W.R., Hoover E., Chen C.C., Wongvipat J., Ku S.Y., Gao D., Cao Z. (2017). SOX2 promotes lineage plasticity and antiandrogen resistance in TP53-and RB1-deficient prostate cancer. Science.

[159] Akamatsu S., Wyatt A.W., Lin D., Lysakowski S., Zhang F., Kim S., Tse C., Wang K., Mo F., Haegert A. (2015). The placental gene PEG10 promotes progression of neuroendocrine prostate cancer. Cell Rep..

[160] Popescu R., Bratu O., Spînu D., Marcu D., Farcaș C., Dinu M., Mischianu D. (2015). Neuroendocrine differentiation in prostate cancer—A review. Rom. J. Mil. Med..

[161] Aparicio A.M., Harzstark A.L., Corn P.G., Wen S., Araujo J.C., Tu S.M., Pagliaro L.C., Kim J., Millikan R.E., Ryan C. (2013). Platinum-based chemotherapy for variant castrate-resistant prostate cancer. Clin. Cancer Res..

[162] Fléchon A., Pouessel D., Ferlay C., Perol D., Beuzeboc P., Gravis G., Joly F., Oudard S., Deplanque G., Zanetta S. (2011). Phase ii study of carboplatin and etoposide in patients with anaplastic progressive metastatic castration-resistant prostate cancer (mCRPC) with or without neuroendocrine differentiation: Results of the French Genito-Urinary Tumor Group (GETUG) P01 trial. Ann. Oncol..

[163] Beltran H., Oromendia C., Danila D.C., Montgomery B., Hoimes C., Szmulewitz R.Z., Vaishampayan U., Armstrong A.J., Stein M., Pinski J. (2019). A phase II trial of the aurora kinase a inhibitor alisertib for patients with castration-resistant and neuroendocrine prostate cancer: Efficacy and biomarkers. Clin. Cancer Res..

[164] Brown L.C., Halabi S., Somarelli J.A., Humeniuk M., Wu Y., Oyekunle T., Howard L., Huang J., Anand M., Davies C. (2022). A phase 2 trial of avelumab in men with aggressive-variant or neuroendocrine prostate cancer. Prostate Cancer Prostatic Dis..

[165] Chin A.I., Ly A., Rodriguez S., Sachdeva A., Zomorodian N., Zhang H., Kim J., Li G., Rettig M., Liu S. (2023). Updated results of a phase Ib single-center study of pembrolizumab in combination with chemotherapy in patients with locally advanced or metastatic small cell/neuroendocrine cancers of the prostate and urothelium. J. Clin. Oncol..

[166] Aggarwal R.R., Zhang J., Zhu X., Monk P., Jones R.J., Linch M.D., Costin D., De Bono J.S., Karsh L.I., Petrylak D.P. (2023). First-in-class oral innate immune activator BXCL701 combined with pembrolizumab in patients with metastatic, castration-resistant prostate cancer (mCRPC) of small cell neuroendocrine (SCNC) phenotype: Phase 2a final results. J. Clin. Oncol..

[167] Mansfield A.S., Hong D.S., Hann C.L., Farago A.F., Beltran H., Waqar S.N., Hendifar A.E., Anthony L.B., Taylor M.H., Bryce A.H. (2021). A phase I/II study of rovalpituzumab tesirine in delta-like 3—Expressing advanced solid tumors. npj Precis. Oncol..

[168] Qiao Y., Choi J.E., Tien J.C., Simko S.A., Rajendiran T., Vo J.N., Delekta A.D., Wang L., Xiao L., Hodge N.B. (2022). Autophagy inhibition by targeting PIKfyve potentiates response to immune checkpoint blockade in prostate cancer. Nat. Cancer.

[169] Sultana Q., Kar J., Verma A., Sanghvi S., Kaka N., Patel N., Sethi Y., Chopra H., Kamal M.A., Greig N.H. (2023). A Comprehensive Review on Neuroendocrine Neoplasms: Presentation, Pathophysiology and Management. J. Clin. Med..

[170] Paisey S.A., Weerasuriya S., Palmer K., White B.E., Srirajaskanthan R., Chandrakumaran K., Ramage J.K. (2022). Primary renal neuroendocrine neoplasms: A systematic literature review, report of four local cases, and original survival analysis of 63 patients from a national registry 2012–2018. J. Neuroendocrinol..

[171] Chu C., Hu C.Y., Batra R., Lin A.Y. (2019). Small cell carcinoma of the kidney: A case report and analysis of data from the Surveillance, Epidemiology, and End Results registry. J. Med. Case Rep..

[172] Shimbori M., Osaka K., Kawahara T., Kasahara R., Kawabata S., Makiyama K., Kondo K., Nakaigawa N., Yamanaka S., Yao M. (2017). Large cell neuroendocrine carcinoma of the kidney with cardiac metastasis: A case report. J. Med. Case Rep..

